# The protective role of conjunctival goblet cell mucin sialylation

**DOI:** 10.1038/s41467-023-37101-y

**Published:** 2023-03-17

**Authors:** Moe Matsuzawa, Tomoaki Ando, Saaya Fukase, Meiko Kimura, Yasuharu Kume, Takuma Ide, Kumi Izawa, Ayako Kaitani, Mutsuko Hara, Eri Nakamura, Anna Kamei, Akira Matsuda, Nobuhiro Nakano, Keiko Maeda, Norihiro Tada, Hideoki Ogawa, Ko Okumura, Akira Murakami, Nobuyuki Ebihara, Jiro Kitaura

**Affiliations:** 1 Atopy (Allergy) Research Center, Juntendo University Graduate School of Medicine, Tokyo, 113-8421 Japan; 2 Department of Ophthalmology, Juntendo University Urayasu Hospital, Urayasu, Chiba 279-0021 Japan; 3 Department of Ophthalmology, Juntendo University Graduate School of Medicine, Tokyo, 113-8421 Japan; 4 Department of Otorhinolaryngology, Juntendo University Graduate School of Medicine, Tokyo, 113-8421 Japan; 5 Laboratory of Molecular and Biochemical Research, Biomedical Research Core Facilities, Juntendo University Graduate School of Medicine, Tokyo, 113-8421 Japan; 6 Research Institute for Diseases of Old Age, Juntendo University Graduate School of Medicine, Tokyo, 113-8421 Japan; 7 Department of Science of Allergy and Inflammation, Juntendo University Graduate School of Medicine, Tokyo, 113-8421 Japan; 8 Department of Immunological Diagnosis, Juntendo University Graduate School of Medicine, Tokyo, 113-8421 Japan; 9 Center for Biomedical Research Resources, Juntendo University Graduate School of Medicine, Tokyo, 113-8421 Japan

**Keywords:** Mucosal immunology, Molecular medicine, Conjunctival diseases

## Abstract

Gel-forming mucins secreted by conjunctival goblet cells have been implicated in the clearance of allergens, pathogens, and debris. However, their roles remain incompletely understood. Here we show that human and mouse conjunctival goblet cell mucins have Alcian blue-detectable sialic acids, but not sulfates in the steady state. Interestingly, Balb/c mouse strain lacks this sialylation due to a point mutation in a sialyltransferase gene, *St6galnac1*, which is responsible for sialyl-Tn synthesis. Introduction of intact *St6galnac1* to Balb/c restores the sialylation of conjunctival goblet cell mucus. Sialylated mucus efficiently captures and encapsulates the allergen particles in an impenetrable layer, leading to the protection of mice from the development of allergic conjunctivitis. Expression of *ST6GALNAC1* and sialyl-Tn is upregulated in humans under conditions with chronic stimuli. These results indicate that the sialylated glycans on the ocular mucins play an essential role in maintaining the conjunctival mucosa by protecting from the incoming foreign bodies such as allergen particles.

## Abstract (web summary)

Conjunctival goblet cells secrete mucins that are heavily glycosylated. Here, the authors show that sialylation of mucins is essential for capturing and encapsulates allergen particles and thus reduces the risk for developing allergic conjunctivitis.

## Introduction

The goblet cell is a specialized epithelial cell type that plays a role in the first-line defense on the wet surfaces of the body including the ocular surface^1–3^. Goblet cells provide hydration and lubrication of the mucosal surface by secreting high molecular weight mucins that are heavily glycosylated^2–4^. The mucus granules containing gel-forming mucins are constitutively exocytosed at the steady state. Upon detection of incoming threats, a burst of release occurs, which entraps the pathogens and debris to facilitate their removal^5–7^. The importance of gel-forming mucins is best exemplified by the fact that deficiency of an intestinal gel-forming mucin, Muc2, leads to spontaneous colitis and enhanced colon cancer development^8,9^. However, in contrast to the intestinal mucosa, which is continuously challenged by luminal bacteria, the roles of single gel-forming mucins in the ocular surface remain elusive especially in specific pathogen-free environments^10,11^. The major gel-forming mucins expressed in the ocular surface include Muc5ac and Muc5b^2^. Although MUC5AC is believed to play a pivotal role in maintaining the rheological properties of the tear film^2^, mice lacking Muc5ac or Muc5b showed only mild, or no dry eye phenotypes^10,11^. Even with the complete loss of conjunctival goblet cells, the dry eye phenotype was mild to moderate^12^. Interestingly, two important phenotypes were observed; an increase of debris in the fornix, which can be attributed to the loss of gel-forming mucins^13^, and a loss of tolerogenic response, which is supported by goblet cell-derived retinoic acid^14^. However, a *Pseudomonas aeruginosa* infection experiment did not prove any protective roles of goblet cells against bacterial infection in the eye^13^ despite the loss of anticipated regulatory effects of gel-forming mucins^15^. Thus, the roles of goblet cell-derived gel-forming mucins in the protection of ocular surface remain incompletely understood.

The mucin proteins harbor repetitive core sequences that are rich in serine and threonine residues, which are known to be extensively O-glycosylated. The major gel-forming mucin in the conjunctiva, MUC5AC, is one of the largest glycoproteins known to date. Its molecular mass reaches more than 20 mDa after glycosylation and multimerization^16^. The O-glycosylated tandem repeat domain is flanked by cysteine-rich domains, and intermolecular disulfide bonding of these domains allows multimerization of the molecules, giving them a gel-like property^4^.

Mucin oligosaccharides account for 50–90% of the molecular mass of the mature glycoprotein. The average glycan mass of the human ocular surface mucin is reported to account for 55%^17^. Most of the glycans are O-glycans, although N-glycans are also found^18–20^. Negative charges of the terminal sialic acid or sulfate group of the glycan chains confer anti-adhesive properties to the mucins^21^. Alterations of mucin glycosylation, including sialylated glycans have been reported in pathological states of ocular surfaces, such as dry eye^22^, contact lens wear pathology^23^, pterygium^24–26^, and ocular rosacea^27^, as well as in the airway^28^, gastrointestinal tract^29^, and cancer^30^. However, whether these alterations are detrimental or protective to the host remains largely unknown, except for cancer O-glycan chains, which have been shown to contribute to biological and pathological consequences^30,31^.

ST6GALNAC is a subfamily of sialyltransferases, which transfers sialic acid with an α2-6 linkage to N-acetylgalactosamine (GalNAc) in O-glycans and glycolipids. Among the six members identified to date in mice and humans, ST6GALNAC1, 2, 4, and to a lesser extent, 3, have an activity on the GalNAc residues in O-linked glycans^32,33^. While ST6GALNAC3 and 4 require sialylation of the acceptor substrate, ST6GALNAC1 and 2 can target non-sialylated structures such as Tn and T antigens^34–36^. In addition, ST6GALNAC1 and 2 have differential preference for their acceptor substrates; ST6GALNAC1 prefers Tn antigen, while ST6GALNAC2 prefers T and sialyl-T antigens in vivo^34,37^. A recent report has shown that the ST6GALNAC1 expressed in the intestine affects sialylation of N-glycans, which plays a role in stabilizing MUC2 mucins against bacterial proteolytic degradation^38^. Although ST6GALNAC1 is reportedly one of the most expressed ST6GALNACs in the human conjunctiva^39^, how ST6GALNAC1 affects MUC5AC and intrinsic properties of the mucus is not known.

The prevalence of ocular allergy has increased in the past decades^40–42^ and is estimated to have reached up to 40–50%, depending on the country^41,42^. The manifestation ranges from less severe but recurrent seasonal and perennial allergic conjunctivitis to severe forms including vernal keratoconjunctivitis (VKC) and atopic keratoconjunctivitis (AKC), which potentially lead to corneal involvement and ultimately to vision loss^42,43^. Alterations of both transmembrane and gel-forming mucin expression have been observed in VKC and AKC^44,45^. More recently, it has been reported that there are increased amounts of sialylated species in the N-glycans present in the tears of VKC and AKC patients^46^. However, the roles of O-glycans and their sialylation in ocular allergy remain unexplored^19,46^.

In this study, we found that goblet cells in the conjunctiva of humans and C57BL/6 J (B6J) mice are Alcian Blue (AB)-positive due to sialylation, while those of Balb/c mice lack it. The major responsible gene was identified as a sialyltransferase, *St6galnac1*. In humans, ST6GALNAC1 and its enzymatic oligosaccharide product, sialyl-Tn, was found to be increased in pathologic conditions under chronic mechanical/inflammatory stimuli. By transferring the intact *St6galnac1* gene to the *St6galnac1*-deficient Balb/c strain, and by generating *St6galnac1*-knockout B6J strain, we investigated the roles of sialylated mucins in vivo.

## Results

### Conjunctival goblet cells of humans and B6J mice, but not those of Balb/c mice, are sialylated in the steady state

**Fig. 1:**
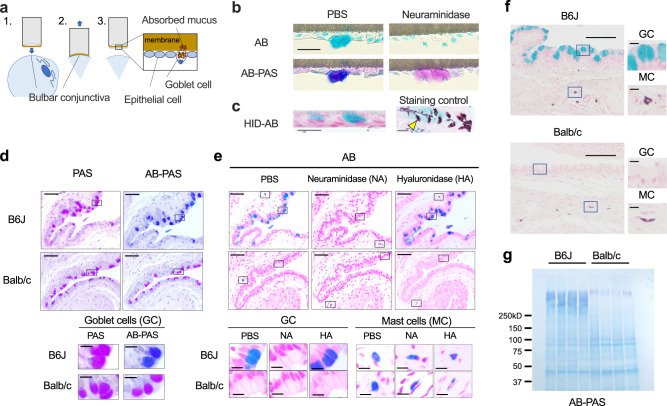
Conjunctival goblet cell mucus of humans and C57BL/6 J mice, but not that of Balb/c mice, is sialylated in the steady state. **a** A schematic diagram of impression cytology. **b** Periodic acid–Schiff (PAS) and Alcian Blue (AB) (pH2.5) staining of the bulbar conjunctival goblet cells from healthy subjects with or without neuraminidase treatment. Bar, 20 μm. **c** High-iron diamine (HID) and AB staining of sulfomucin of the human conjunctival goblet cells. Staining control: mouse colon. The arrowhead indicates an example of HID-positive (brown) goblet cell. Bar, 20 μm. PAS and AB-PAS (**d**), or AB staining (**e**) of mouse conjunctiva. Bar 50 μm. Inset: Bar, 10 μm. Tissue sections were treated with neuraminidase (NA) or hyaluronidase (HA) before staining in (**e**). GC, goblet cells; MC, mast cells. **f** HID-AB staining of the mouse conjunctivas. Bar, 100 μm. Inset: Bar, 10 μm. **g** AB-PAS staining of membrane-transferred conjunctival swab proteins. Data are representative of at least two independent experiments (**b–g**). Source data are provided as a Source Data file.

Previous studies have demonstrated that Alcian Blue (AB) stains human, rat, and mouse conjunctival goblet cells differentially or simultaneously with Periodic acid-Schiff (PAS) staining depending on the disease state and age^47–50^. Because AB stains acidic residues such as sialic acid, hyaluronic acid, and sulfate groups on the glycoproteins, we used AB staining in attempt to elucidate the responsible acidic residues in the goblet cell mucus granules. Steady state human conjunctival goblet cells were positive for AB, but negative for high iron diamine (HID), which stains sulfate groups (Fig. 1a–c). In addition, desialylated conjunctival goblet cells became negative for AB, suggesting that the AB positivity of human goblet cells are derived from their sialic acid (Fig. 1b). However, goblet cells of B6J and Balb/c mice were stained differently; B6J goblet cells were positive for both AB and PAS, while Balb/c goblet cells were positive for PAS staining only (Fig. 1d). Neuraminidase, but not hyaluronidase treatment, abolished the AB staining of the B6J goblet cells, indicating that sialylated glycans are the major substances stained by AB, and these are present only in the B6J goblet cells (Fig. 1e). Goblet cells of both strains were negative for HID (Fig. 1f). Of note, mast cell AB staining was retained after neuraminidase and hyaluronidase treatments, consistently with the positive HID staining (Fig. 1e, f). AB-PAS staining of the swab extract of the conjunctiva revealed that the major AB-positive (and negative in Balb/c) PAS-positive protein has a high molecular weight, indicating that the mucins are the major substances that harbor sialylated glycan stained in conjunctival goblet cells (Fig. 1g).

### Aberrant splicing disrupts the sialyl motif of St6galnac1 in Balb/c mice

**Fig. 2:**
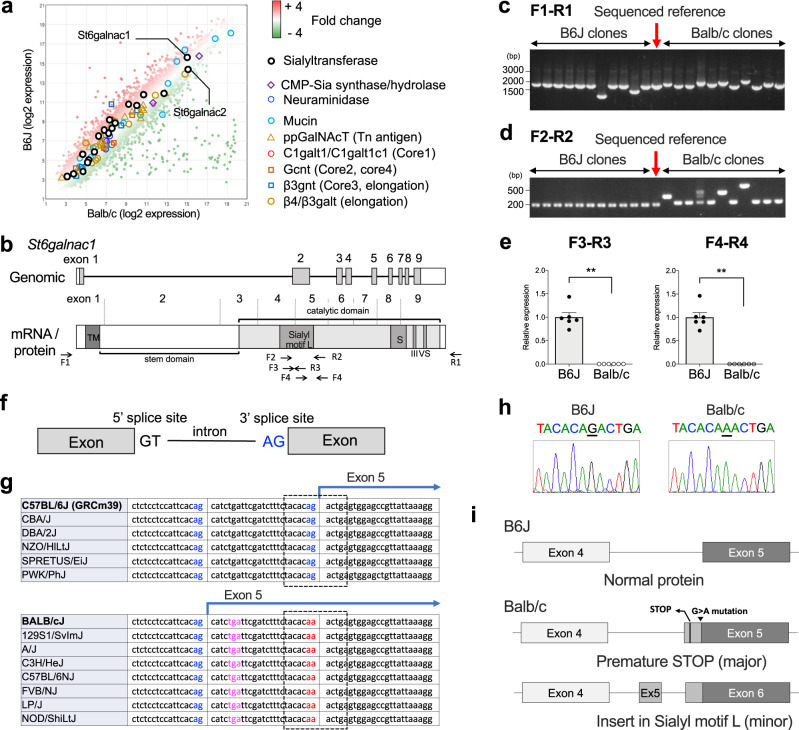
Aberrant splicing disrupts sialyl motif of St6galnac1 in Balb/c mice. **a** Pooled total RNA from three eyes for each strain was subjected to microarray analysis. CMT-Sia, cytidine-5ʹ -monophospho-N-acetylneuraminic acid; ppGalNAcT, peptidyl-N-acetylgalactosaminyl-transferases; Gcnt, glucosaminyl (N-acetyl) transferase; β3gnt, β3-GlcNAc-transferase; galt, galactosyltransferase. **b** Schematic representation of *St6galnac1* gene and its product. The catalytic domain has four conserved sialyl motifs (L, S, III, VS). Priming sites for PCR used in (**c–e**) are shown. TM, transmembrane domain. Colony PCR (**c**) and nested PCR (**d**) of cloned St6galnac1. Data are representative of two independent experiments. **e** Quantitative PCR for intact sialyl motif L. *n* = 6 for each strain. **p < 0.01 (*p* = 0.0022 for F3-R3 and *p* = 0.0022 for F4-R4) by two-tailed Mann–Whitney test. Data represents mean ± S.E.M. Data are representative of two independent experiments. **f** Schematic representation of splicing sites. **g** Strain comparison of 3ʹ splice site sequences available at Ensembl database^52^. Blue, potential 3ʹ splice sites; Red, disabled 3ʹ splice site; Magenta, premature stop codon. The range of Sanger sequencing shown in (**h**) is circled with a dotted line. **h** Sanger sequencing of 3ʹ splice site of intron before exon 5. **i** Schematic representation of exon usages of cloned *St6galnac1*. Source data are provided as a Source Data file (**c**, **d**, **e**).

In search of the genes responsible for differential sialylation between B6J and Balb/c strains, we compared the expression profiles of the conjunctival tissues using gene microarray. To our surprise, there were no genes related to sialylation showing all-or-none expressions between the two strains (Fig. 2a). Instead, high expression of a few sialyltransferases, including *St6galnac1*, was observed in both the strains (Fig. 2a). This indicated that qualitative, rather than quantitative, differences led to the all-or-none phenotype of sialylation.

To examine whether there are any functional differences in St6galnac1 which is expressed highest in the conjunctiva of the two strains, we cloned *St6galnac1* mRNA expressed in the conjunctiva. St6galnac1 is a type 2 transmembrane protein residing in the Golgi apparatus, that is comprised of an N-terminus transmembrane domain followed by a long stem and a catalytic domain (Fig. 2b). The catalytic domain has four regions called “sialyl motifs”, which are conserved among all classes of sialyltransferases^51^. The amplicon sizes of the cloned *St6galnac1* were highly variable in Balb/c mice, compared to those of the relatively uniform B6J clones (Fig. 2c). Furthermore, the lengths of the Balb/c amplicons corresponding to the site of Sialyl motif L were different from the known RefSeq sequence (Fig. 2d), suggesting a difference in the coding sequences. In fact, two pairs of quantitative-PCR primers detected no mRNA coding intact Sialyl motif L in the Balb/c conjunctiva (Fig. 2e). Examination of genomic and cloned cDNA sequences revealed that a point mutation at the 3ʹ splice site (G > A) before exon 5 led to aberrant splicing in Balb/c mice (Fig. 2f–i). Comparison of publicly available mouse genomic sequences^52^ revealed that the G > A mutation was found across several mouse strains (Fig. 2g). Most of the Balb/c clones resulted in the premature stop codon, with loss of most of the catalytic domain (Fig. 2g,i). We searched for potentially functional Balb/c clones that are derived from in-frame alternative splicing and found a rare clone with an extra exon that results in an insertion in the Sialyl motif L amino acid sequence (Fig. 2i).

### Defective St6galnac1 activity in Balb/c mice

**Fig. 3:**
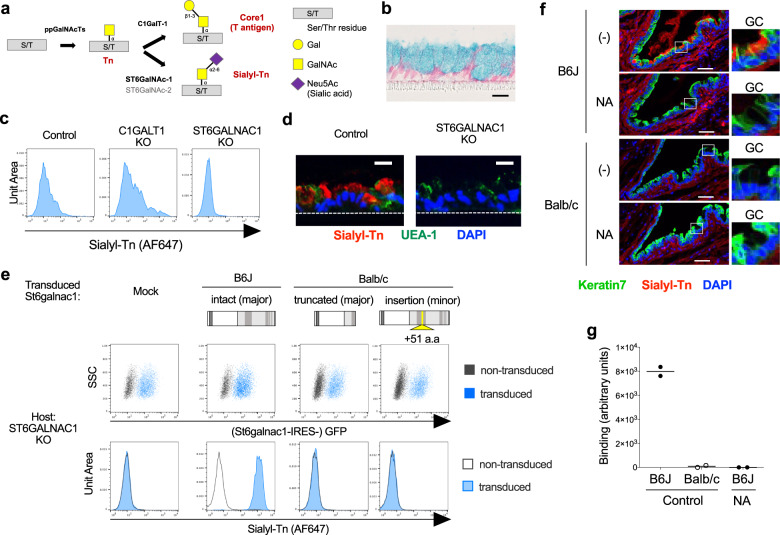
Defective St6galnac1 activity in Balb/c goblet cells. **a** Biosynthetic pathways of Tn, Core1, and sialyl-Tn. S/T, serine or threonine residue. **b** AB and Kernechtrot staining of differentiated HT29-MTX-E12 (E12) cell line. Bar, 20 μm. Polyclonal knockout (KO) of *C1GALT1* or *ST6GALNAC1* in E12 cells by genome editing affects sialyl-Tn expression assessed by FACS (**c**) and immunostaining (**d**). UEA-1, Ulex Europaeus Agglutinin I. Bar, 20 μm. **e** *ST6GALNAC1*-KO E12 cells were transduced with cloned *St6galnac1* sequences. Expression levels of sialyl-Tn and GFP in transduced (blue histogram) and un-transduced (gray empty histogram) cells were evaluated by FACS. a.a, amino acids. **f** B6J and Balb/c conjunctivas were stained with a monoclonal antibody against sialyl-Tn (clone MLS132) with or without neuraminidase treatment. GC, goblet cells. Bar, 50 μm. **g** Swab extract of conjunctival sac (*n* = 2 for each condition) was subjected to neuraminidase or BSA (control) treatment before coating onto the plate. Sialyl-Tn was detected by anti-sialyl-Tn antibody. Source data are provided as a Source Data file. Data are representative of two independent experiments.

To test the enzymatic activity of cloned St6galnac1 isoforms to synthesize sialyl-Tn (Fig. 3a), we employed a human goblet cell line HT29-MTX-E12 (E12)^53^. E12 cells differentiated into mature goblet cells, by approximately two weeks of culture after becoming confluent (Fig. 3b). E12 was found to expresses a small amount of sialyl-Tn, which was enhanced upon CRISPR/Cas9-mediated knockout of core-1 synthase (*C1GALT1*), and abrogated by knockout of *ST6GALNAC1* (Fig. 3c and Supplementary Fig. 1). The goblet cell mucus granules were positive for sialyl-Tn, suggesting that the sialyl-Tn is present on the mucins (Fig. 3d). Retroviral transduction of intact full-length *St6galnac1*, but not the Balb/c-derived clones, dramatically increased sialyl-Tn expression (Fig. 3e). Although lectins known to bind some sialylated glycan structures did not bind B6J conjunctival goblet cells (Supplementary Fig. 2a,b), sialyl-Tn was detected in the B6J conjunctival goblet cells and released mucus, but not in the Balb/c goblet cells (Fig. 3f). Consistently with this, the conjunctival swab from Balb/c mice did not have any detectable sialyl-Tn (Fig. 3g). These results indicate that only the St6galnac1 protein derived from the intact B6J transcript is functional, while those from Balb/c transcripts are not.

### Expression of intact St6galnac1 restores sialylation of goblet cell mucins in Balb/c mice

**Fig. 4:**
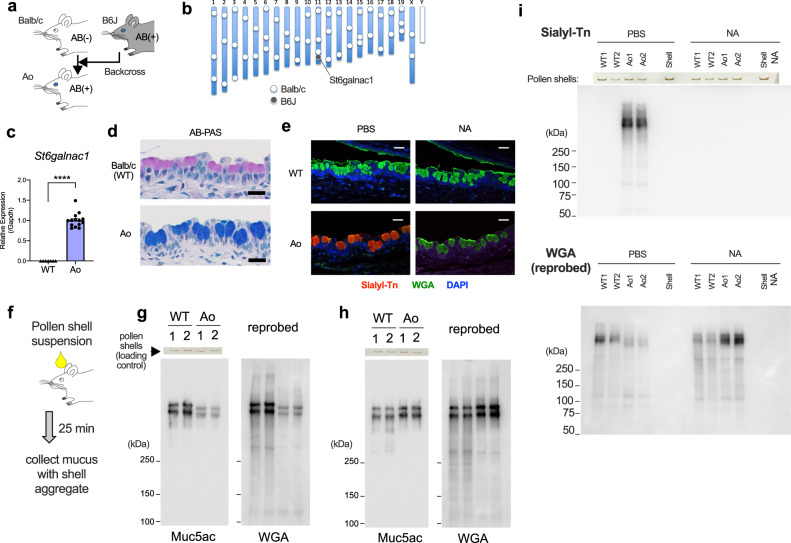
Expression of intact St6galnac1 restores Sialyl-Tn expression in conjunctival goblet cell mucins in vivo. **a** Schematic representation of backcrossing of B6J-derived intact *St6galnac1* gene to Balb/c mice. **b** Chromosomal distribution of PCR-confirmed strain-specific markers in Ao mice. **c** Intact *St6galnac1* expression in the conjunctiva. *n* = 7 for WT and *n* = 14 for Ao mice. ****p < 0.0001 by two-tailed Mann–Whitney test. Error bar represents S.E.M. **d** AB-PAS staining of the conjunctiva. Bar, 20 μm. **e** Immunostaining of sialyl-Tn in conjunctiva with or without neuraminidase treatment. WGA, wheat germ agglutinin Bar, 20 μm. **f** A schematic diagram of secreted mucus collection. Western blotting of the secreted mucus with (**h**, **i**) or without (**g**, **i**) neuraminidase treatment. The photographs of pollen shell amount left in the wells of polyacrylamide gels after the electrophoresis are also shown as loading controls (**g–i**). Data are representative of at least two independent experiments (**d**, **e**, **g–i**). Source data are provided as a Source Data file (**c**, **g**, **h**, **i**).

To assess the physiological role of St6galnac1, we introduced B6J-type intact *St6galnac1* allele to the Balb/c strain by backcrossing (Fig. 4a, b and Supplementary Fig. 3). Forty eight simple sequence length polymorphism markers were genotyped^54^ to confirm the replacement of chromosomal arms other than the *St6galnac1* locus, after nine generations of backcrossing (Fig. 4b). The conjunctival tissue of fully backcrossed mice, termed Ao mice, expressed sialyl-motif-intact *St6galnac1* (Fig. 4c) and had AB-positive goblet cells (Fig. 4d). The presence of sialyl-Tn was confirmed by immunostaining (Fig. 4e). However, there was no difference in the amount of tear volume between WT and Ao mice (Supplementary Fig. 4). To assess the properties of mature mucus proteins, we stimulated mucus secretion by applying ragweed pollen shells essentially devoid of proteins and collected them as the shell aggregates (Fig. 4f). Most of the WGA-reactive glycoprotein was found to be Muc5ac, with different mobilities between WT and Ao mice (Fig. 4g). The mobilities were the same after neuraminidase treatment, indicating that the difference may be attributable to negative charges of the sialic acids (Fig. 4h). The sialylation was represented by the existence of sialyl-Tn (Fig. 4i). These results demonstrate that St6galnac1 is sufficient for the synthesis of sialic acid-capped glycans, including sialyl-Tn, in murine conjunctival goblet cells.

### Sialylated mucins encapsulate pollens with an impenetrable layer

**Fig. 5:**
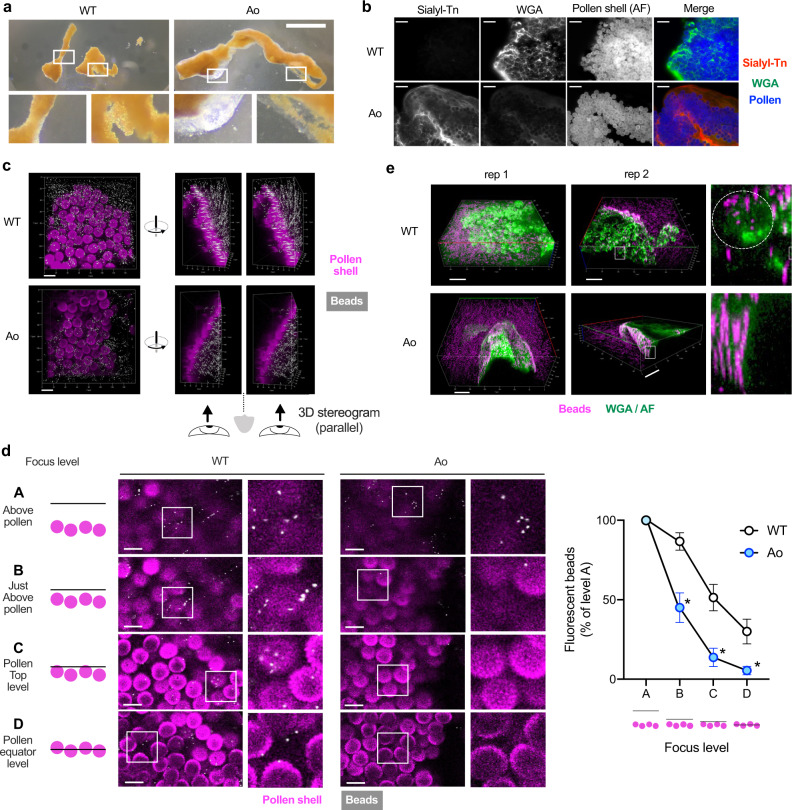
Sialylated mucins encapsulate pollens with an impenetrable layer. **a** Representative pictures of pollen shell-mucus aggregates 25 min after challenge. Bar, 1 mm. See also Supplementary Fig. 5 for more examples. **b** Immunostaining of pollen shell aggregates retrieved from the conjunctival sac. AF, autofluorescence. Bar, 50 μm. **c** The pollen shell aggregates from indicated strain were soaked in the fluorescent beads-suspended PBS. Parallel-view 3D stereogram of the side-view is also shown. Bar, 20 μm along the closest edge. **d** The bead distribution was enumerated at various focus levels. Bar, 20 μm. *n* = 6 for WT and *n* = 7 for Ao mice. *p < 0.05 (adjusted *p* = 0.0101, 0.0101, and 0.0233 for levels B, C, and D) by two-tailed Two-way ANOVA with Holm-Sidak’s multiple comparisons. Focus level A is excluded from the statistical analysis. *n* = 6 for WT and *n* = 7 for Ao mice. Error bar represents S.E.M. Source data are provided as a Source Data file. See also Supplementary Fig. 6. **e** The pollen shell aggregates were soaked in fluorescent beads-suspended PBS containing WGA. The detail of the white-squared area is also shown. The white dotted circle indicates a pollen shell. Bar, 50 μm along the closest edge; rep, representative pollen shell aggregate. Data are representative of at least two independent experiments (**a–e**).

To investigate the functions of sialylated mucins, we examined the pollen aggregates under a microscope. Intriguingly, mucus from Ao mice, but not that from WT Balb/c mice, encapsulated the pollen aggregate with a layer of gel-like structure (Fig. 5a and Supplementary Fig 5). The layer was sialyl-Tn-positive, but the immunostaining intensity was lower compared to that of the mucus inside the aggregate, implying that the water content is higher in the gel-like layer (Fig. 5b). To assess the penetrability of the mucus layer, we took an inverse penetration approach; the mucus-encapsulated pollen aggregates were soaked into the fluorescent particle suspension, and the space accessible to the fluorescent particles was assessed. In WT Balb/c, the pollen shell surface was readily accessible to the particles with a diameter of 0.5 μm (Fig. 5c, d and Supplementary Fig. 6). However, in Ao mice, most of the particles did not reach the shell surface (Fig. 5c, d and Supplementary Fig. 6). Furthermore, additional staining with WGA revealed the difference of surface texture of the mucus; Ao mucus completely covered the pollen aggregate, while the WT mucus did not hide the pollen balls (Fig. 5e). Given that the sizes of most of the bacteria are in the similar order (0.5–2 μm) to the fluorescent particles used, and so do the subpollen particles that carry allergenic proteins (0.5–4.5 μm)^55^, the sialylated mucins may have a role in quarantining potentially harmful particles, as a first-line of defense on the ocular surface.

### Sialylated mucins efficiently forms large aggregates of pollen shells

**Fig. 6:**
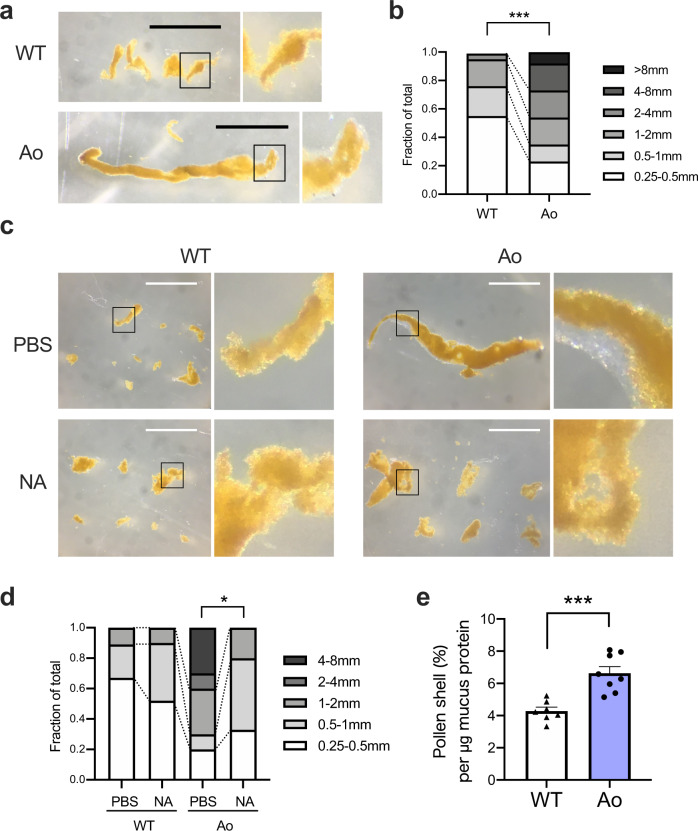
Sialylation is required for the efficient entrapment of pollen shells. **a** Representative picture of pollen shell-mucus aggregates from one eye. Bar, 1 mm. **b** Frequencies of aggregates with indicated lengths. Pooled data from 8 eyes each. ***p < 0.001 (*p* = 0.0009) by two-tailed Fisher’s exact test for the ratio of fractions <1 mm and ≧1 mm. **c** The pollen shells were instilled into eyes with or without 5 U/mL neuraminidase. Bar, 1 mm. **d** Frequencies of aggregates with indicated lengths. Pooled data from 4 eyes each. *p < 0.05 (*p* = 0.0344) by two-tailed Fisher’s exact test for the ratio of fractions <1 mm and ≧1 mm. **e** The pollen shell amount in the aggregate was divided by the amount of mucus protein in the same aggregate. *n* = 7 for WT and *n* = 8 for Ao mice. ***p < 0.001 (*p* = 0.0004) by two-tailed Welch’s *t* test. Error bar represents S.E.M. Data are representative of at least three independent experiments (**a–e**). Source data are provided as a Source Data file (**b**, **d**, **e**).

In addition to the gel-like layer, we noticed that secreted mucus from Ao mice formed significantly larger aggregates compared to that from WT Balb/c mice (Figs. 5a, 6a, b and Supplementary Fig. 5). The aggregate sizes were decreased when the pollen shells were administered together with neuraminidase (Fig. 6c, d). Gel-like layer also disappeared by sialic acid removal (Fig. 6c). Furthermore, significantly larger amount of pollen shells were captured by the same amount of mucus protein in Ao mice, as compared to those captured in WT Balb/c mice (Fig. 6e).

### Chronic stimulatory conditions upregulate sialyl-Tn and ST6GALNAC1 in humans

**Fig. 7:**
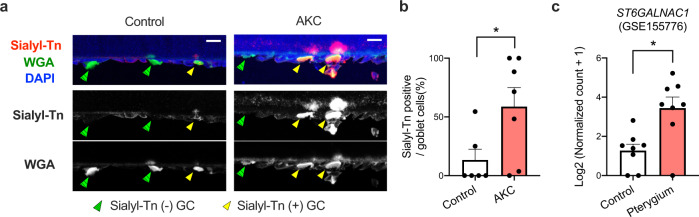
Sialyl-Tn or *ST6GALNAC1* is upregulated in chronic stimulatory conditions in humans. **a** Representative immunostaining of impression cytology specimens. GC, goblet cells. Bar, 20 μm. **b** Frequencies of sialyl-Tn-positive goblet cells in impression cytology specimens from control (*n* = 6) and AKC (*n* = 7) subjects. *p < 0.05 (*p* = 0.0443) by two-tailed Mann–Whitney test. Error bar represents S.E.M. **c** *ST6GALNAC1* expression in an RNA-seq dataset (GSE155776) of pterygium (*n* = 8) and control (*n* = 8) biopsies. *p < 0.05 (*p* = 0.0103) by two-tailed Mann–Whitney test. Error bar represents S.E.M. Source data are provided as a Source Data file (**a**, **b**).

Since sialylated mucins could play a protective role by capturing and quarantining incoming foreign bodies to the eyes, it may be increased in conditions with chronic mechanical and/or inflammatory stimuli. Indeed, we found increase of sialyl-Tn-positive goblet cells in the conjunctiva of AKC patients (Fig. 7a, b). In addition, analysis of a published dataset^56^ revealed that *ST6GALNAC1* expression levels were significantly increased in the biopsies of pterygium, a relatively common proliferative ocular surface disorder associated with UV exposure and inflammation^57^, compared to those in healthy conjunctiva (Fig. 7c).

### St6galnac1 protects mice from pollen-induced allergic conjunctivitis

**Fig. 8:**
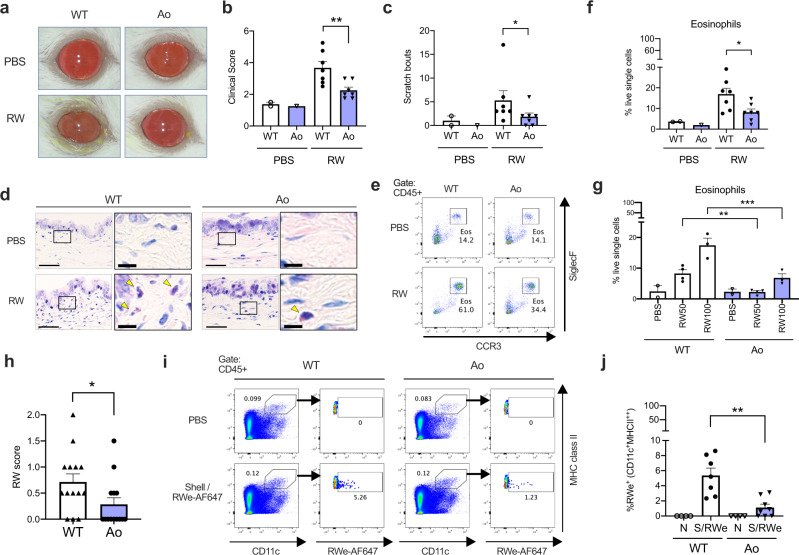
St6galnac1 protects mice against allergic conjunctivitis. **a** Clinical appearance of PBS- or ragweed pollen (RW)-challenged eyes. **b** Clinical scores. *n* = 2, 1, 7, and 7 for PBS-WT, PBS-Ao, RW-WT, and RW-Ao conditions, respectively (**b**, **c**, **f**). **p < 0.01 (*p* = 0.0093) by two-tailed Mann–Whitney test. **c** Scratch bouts. *p < 0.05 (*p* = 0.0449) by two-tailed Mann–Whitney test. **d** Giemsa staining of the conjunctiva. Arrowheads indicate eosinophils. Bar, 50 μm. Inset: Bar, 10 μm. **e** Eosinophil frequency among CD45 + cells in the conjunctiva. Eosinophil frequencies in live single cells after challenges of 100 mg/mL (**f**) or indicated amount (mg/mL) (**g**) of RW pollen or PBS. *p < 0.05 (*p* = 0.0379) by two-tailed Mann–Whitney test (**f**). **p < 0.01 (adjusted *p* = 0.0091, WT vs Ao in RW 50 mg/mL condition), ***p < 0.001 (adjusted *p* = 0.0006, WT vs Ao in RW 100 mg/mL condition) by two-tailed ANOVA with Holm-Sidak’s multiple comparisons test (**g**). **h** RW scores represent the amount of remaining pollen in the conjunctival sac 30 min after pollen challenges. *n* = 14 for each strain. *p < 0.05 (*p* = 0.0325) by two-tailed Mann–Whitney test. **i**, **j** Accumulation of migratory dendritic cells (CD11c^+^MHC class II high) to the draining lymph nodes 12 h after instillation of pollen shells and RW extract labeled with AF647. FACS gating for pooled data (**i**) and enumeration (**j**) are shown. N, PBS-instilled; S/RWe, pollen shell and AF647-labeled RW extract. PBS-instilled data were used for setting AF647 gate and therefore excluded from statistical analysis. *n* = 4, 7, 4, and 8 for WT-N, WT-S/RWe, Ao-N, and Ao-S/RWe conditions, respectively. **p < 0.01 (*p* = 0.0037) by two-tailed Mann–Whitney test. Data are representative of four (**a–h**) or three (**i**, **j**) independent experiments. Error bar represents S.E.M. (**b**, **c**, **f**, **g**, **h**, **j**) Source data are provided as a Source Data file (**b**, **c**, **f**, **g**, **h**, **j**).

Finally, we investigated whether sialylated mucins are functionally protective in vivo. We applied a well-established murine model of ragweed (RW) pollen-induced conjunctivitis to WT Balb/c and Ao mice (Supplementary Fig. 7a). Ao mice showed significantly ameliorated clinical manifestations (Fig. 8a–c). In addition, the eosinophil accumulations were attenuated in Ao mice (Fig. 8d–g and Supplementary Fig. 8). Dose-response analysis showed that the presence of intact St6galnac1 increases the antigen dose threshold for inducing eosinophil accumulation (Fig. 8g). Although the humoral responses were similar after immunization (Supplementary Fig. 7b–d), RW-specific IgE was elevated after pollen challenges in WT mice, but not in Ao mice, suggesting that the sialylated mucins can reduce the antigen responses (Supplementary Fig. 7b–d). Indeed, the amounts of pollens remaining in the conjunctival sac of Ao mice after challenges were significantly smaller than those of WT Balb/c mice, suggesting that the sialylated mucus can efficiently remove the pollen particles compared to the non-sialylated one (Fig. 8h and Supplementary Fig. 9). Furthermore, the fraction of the migrating dendritic cells ingesting the instilled allergen was found to be smaller in Ao mice than in WT Balb/c mice in the draining lymph node (Fig. 8i, j). These results suggest that the sialylated mucins play an essential role in protecting the ocular surface from incoming foreign bodies including allergens (Supplementary Fig. 10).

To further confirm the role of sialylated mucins in vivo, we generated *St6galnac1* knockout (KO) mice on the B6J background using CRISPR-Cas9 technique (Supplementary Fig. 11a). As expected, conjunctival goblet cells of *St6galnac1*-KO mice were completely negative for sialyl-Tn, indicating the successful disruption of *St6galnac1* gene (Supplementary Fig. 11b). However, there were some sialyl-Tn-negative AB-positive goblet cells left in the *St6galnac1* KO conjunctiva (Supplementary Fig. 11b). The AB-stained substance was sialic acid, as there were no AB-positive cells after neuraminidase treatment (Supplementary Fig. 11b). This indicated that another sialyltransferase activity was constitutively operative, or expressed as a compensatory mechanism, in a minor subset of B6J goblet cells, and it was inactive in Balb/c mice. As we found some missense mutations in Balb/c sialyltransferases other than *St6galnac1* (Supplementary Table 2), these might affect their functionality. Interestingly, partial formation of gel-like layer was observed around the pollen aggregates from *St6galnac1* KO mice, suggesting that partially expressed sialylated glycan other than sialyl-Tn may contribute to the gel-like layer formation (Supplementary Fig. 11c). However, the lengths of the pollen aggregates were smaller (Supplementary Fig. 11d), and the eosinophilic inflammation upon induction of allergic conjunctivitis was exacerbated in *St6galnac1* KO mice compared to those in WT mice (Supplementary Fig 11e,f). These results suggest that the decrease in sialylation of mucins in *St6galnac1* KO mice results in less-protective phenotypes, illustrating the protective function of sialylated mucins.

## Discussion

In the present study, we report the protective role of sialylated mucins present in conjunctival goblet cells. The humans and B6J mice have sialylated mucins in conjunctival goblet cells, while Balb/c mice lacked this sialylation. The difference was largely due to a point mutation at the 3ʹ splice site of a sialyltransferase, *St6galnac1*, which we found is highly expressed in the mouse conjunctiva, similarly to the human conjunctiva^39^. In Balb/c strain, loss of St6galnac1 activity, in addition to another sialyltransferase activity found in a minor population of conjunctival goblet cells, led to loss of sialylation on goblet cell mucins. Expressing intact St6galnac1 in Balb/c mice restored this sialylation, which was required for the efficient entrapment and encapsulation of the allergen into a gel-like layer, which was impenetrable to sub-micrometer particles. Human conjunctiva was found to express increased amount of *ST6GALNAC1* and sialyl-Tn under chronic stimuli. Finally, the pollen-induced allergic conjunctivitis was ameliorated by expressing sialylated mucins, with enhanced removal of the pollen particles.

The apical surface barriers of mucosal tissue may consist of several layers, depending on the location. For instance, the intestinal surface of the ileum is protected by a single layer of penetrable mucus layer, under which is a rigid glycocalyx on the apical side of the epithelial cells^7,58^. In the distal colon, where the bacterial load is highest in the intestine, there are two mucus layers; the outer layer serves as a habitat for microbiota, while the inner layer is a separating border that is free of bacteria^7,59^. The tear film of the ocular surface is composed of three layers; the lipid layer that prevents water evaporation, the aqueous/mucoaqueous layer that contains soluble and gel-forming mucins, and the glycocalyx that serves as a physical barrier^2,60^. Recently, a new model of the distal colon mucus layers has been proposed, in which the two mucus layers are encapsulating the feces, rather than lining the epithelial surface, to limit the bacterial contact with the epithelial surface^61,62^. For this to be functional, the proximal colon-derived O-glycosylated mucus was required^61^. Importantly, allowing bacterial contact to the epithelium would expose the host to the risk of inflammation, even in the case of commensals^63^. Our observation that the released mucus encapsulates the particulate allergens suggests that the goblet cells enforce two strategies to protect the ocular surface by gel-forming mucins: maintaining the stability of the tear film at steady state, and capturing and quarantining the large foreign bodies to enhance their removal in emergency. Our results indicate that sialylation of mucins is necessary for the latter to be effective.

The molecular sieve of the bacteria-impenetrable inner layer of colon mucus is reported to be denser compared to that of the outer layer^7,64^. The penetrability is associated not only with the thickness, but also with the glycan component^63^. At the protein or small compound level, the mucins can inhibit drug diffusion^65^, but the retention of IgA is enhanced by the presence of the mucus layer^66^, which is beneficial for the protection of mucosal surfaces^67^. Interestingly, both the outer and inner layers of the colon mucus, and the penetrable layer of the ileum are composed of the same mucin, Muc2, suggesting that the same gene can produce mucus layers with different physical properties^7^. In line with this, loss of Core-1 synthase C1galt1 in the intestinal epithelial cells has been reported to impair the integrity of the mucus layer^63,68^. Interestingly, C1galt1-deficient colonic goblet cells have increased Tn antigen, which implies a reduction of the total negative charges of sialylation or sulfation on the mucin glycans^68^. In the conjunctiva, our results indicated that St6galnac1-dependent sialylation can provide negative charges to the mucin glycans as observed by the increased mobility on SDS-PAGE. In addition, sialylation increased pollen-capturing efficiency, and the impenetrable capsule layer was missing without mucus sialylation. Thus, we propose that the sialylation is one of the most important factors that regulate Muc5ac gel-forming mucin functions in the conjunctiva.

We previously reported that the pollen shells play an essential role in conjunctival local sensitization as well as in the elicitation phase of the allergic conjunctivitis^69^. In addition, raw ragweed pollens have been shown to release sub-pollen particles upon being soaked in water, which in turn enhance the allergic inflammation^55^. Our observation that the pollen particles can be encapsulated with a sialylated impenetrable layer suggest that the layer may have a role in protecting epithelial cells from mechanical damage and in preventing the microparticles to be released. As such, the enhanced removal of pollen particles and reduced migration of allergen-bearing DCs to the lymph nodes was observed in mice capable of producing sialylated mucins. Further investigation is required to elucidate the consequences of sialylation on other mucus properties such as stability against various proteases and permeability to the proteins and small molecules.

In summary, our study elucidated the role of sialylated goblet cell mucins in providing protection against foreign bodies such as allergen particles.

## Methods

### Mice

B6J and Balb/c mice were bred in the animal facility of Juntendo University or purchased from Sankyo Labo Service Corporation (Tokyo, Japan). Ao mice and *St6galnac1* knockout mice were generated as described below. All mice used in this study were 5-9 weeks old. Male and female mice were equally used. Mice were fed ad libitum with a standard diet (Charles River formula-1 (CRF-1), Catalog number (Cat.#) 2108200, Oriental Yeast, Japan) and maintained in plastic cages containing wood chip in an air-conditioned and light-controlled animal room (23 ± 1 C, 55 ± 5% humidity, 12 h light/ 12 h dark).

### Generation of Ao mouse

Ao mice were generated by backcrossing B6J mice to Balb/c mice. See Supplemental Fig. 3 and Supplemental Table 1 for the detail of genotyping information. Simple sequence length polymorphism regions (D1Mit231, D1Mit218, D1MIt150, D2Mit149, D2Mit328, D2Mit265, D3Mit164, D3Mit189, D3Mit19, D4Mit193, D4Mit203, D5Mit233, D5Mit161, D6Mit138, D6Mit8, D6Mit36, D7Mit267, D7Mit101, D8Mit178, D8Mit211, D9Mit8, D9Mit18, D10Mit213, D10Mit230, D11Mit226, D11Mit39, D11Mit333, D12Mit12, D12Mit114, D12nDs2, D13Mit16, D13Mit213, D14Mit60, D14Mit194, D15Mit265, D15Mit80, D15Mit34, D16Mit131, D16Mit140, D17Mit133, D17Mit123, D18Mit222, D18Mit184, D19Mit68, D19Mit19, D19Mit1, DXMit48, DXMit153) were genotyped to confirm the replacement of chromosomes^54^.

### Generation of *St6galnac1*-knockout mice

*St6galnac1*-knockout mice were generated by CRISPR/Cas9 system^70^ in our facility. Ribonucleoprotein was assembled using Alt-R S.p. HiFi Cas9 Nuclease V3 (Integrated DNA technologies, USA), Alt-R CRISPR-Cas9 tracrRNA (Integrated DNA technologies), and Alt-R CRISPR-Cas9 crRNA (Mm.Cas9.ST6GALNAC1.1.AB,/AlTR1/rArUrArCrArCrUrUrGrGrArGrUrArCrCrCrArGrUrGrUrUrUrUrArGrArGrCrUrArUrGrCrU/AlTR2/, Integrated DNA technologies) and was microinjected into the cytoplasm of fertilized 1-cell eggs at the pronuclei stage from C57BL/6 J female mice (Charles River Laboratories Japan, Inc., Kanagawa Japan) superovulated by intraperitoneal injection of PMSG followed by hCG at an interval of 48 h and mated overnight with C57BL/6 J male mice (Charles River Laboratories Japan, Inc., Kanagawa Japan). Microinjections were performed by micromanipulators (Leica, Wetzlar, Germany) with a PMM-150 FU piezo-impact drive unit (Prime Tech Inc., Ibaragi, Japan) using a blunt-ended mercury-containing injection pipette with approximately 6 μm of inner diameter^71^. After microinjection, 2-cell stage embryos, cultured in modified Whitten’s medium^72^ (Mitsubishi Kagaku Iatron Inc., Tokyo, Japan) for approximately 24 h and developed from fertilized 1-cell eggs, were transferred into the oviducts of pseudopregnant ICR females (Charles River Laboratories Japan, Inc., Kanagawa, Japan) at 0.5 dpc.

Mutations were evaluated by Sanger sequencing of PCR product from genomic DNA. Targeted region was PCR amplified using Q5 High-Fidelity DNA Polymerase (New England Biolabs, USA) and following primers: Forward: 5ʹ-GCTGCTTCAGAGCTGAGG-3ʹ and reverse: 5ʹ-AGCCCATATCTACAAGCAACG-3ʹ.

The product was cleaned using NucleoSpin Gel and PCR Clean-up (MACHEREY-NAGEL, Germany) kit following manufacturer’s instructions. Sanger sequence was performed using the reverse primer as the sequencing primer. Obtained knockout lines were crossed with wild-type C57BL/6 J mice to segregate the knockout alleles. The heterozygous pups were genotyped by Sanger sequencing, and the pups harboring the same mutation was crossed to generate homozygous knockout mice.

### Allergic conjunctivitis models

The experimental allergic conjunctivitis models were induced as previously described^69^. For Balb/c background strains, each foot pad of Balb/c or Ao mice received 50 μg of RW pollens emulsified in 50 μL of Imject Alum Adjuvant (ThermoFisher Scientific) on day 0. On day 14, 0.1 mg of RW pollens suspended in 200 μL of PBS was injected intraperitoneally. From day 28, each conjunctival sac received 2.5 μL of 100, 50, or 0 mg/mL RW pollen suspension once a day for 4 consecutive days (Supplementary Fig. 7a). For B6J background strains, the local sensitization model was used^69^. Each conjunctival sac received 2.5 μL of 200 mg/mL RW pollen suspension once a day for 5 days per week for 3 weeks. 3 days later, the last challenge was performed at the same dose (Supplementary Fig. 11e).

### Evaluation of allergic conjunctivitis model

Mouse eyes were scored for the signs of immediate hypersensitivity responses 30 min after the topical challenges of RW pollen suspension, according to previously published criteria^69^. Conjunctival redness, chemosis, tearing, and eyelid edema were graded as 0 (absent), 1 (mild), 2 (moderate), or 3 (severe) by a person blind to the mouse identity. Signs that appeared to be in the middle of the grades were scored in increments of 0.5. Scores of the left and right eyes were averaged to calculate the final clinical score assigned to each mouse. The remaining ragweed pollen amount was graded from 0 to 2, as shown in Supplementary Fig. 9 and assigned to each eye. Scratching behavior with hind legs were also counted for 30 minutes. Mice were sacrificed 24 h after the last challenge and subjected to analysis.

### Cell culture

HT29-MTX-E12 (E12) cell line was obtained from European Collection of Authenticated Cell Cultures (ECACC, UK). Cells were cultured in DMEM supplemented with 10% fetal calf serum, 1% nonessential amino acids and 1% L-glutamine. For differentiation, cells were seeded on Millicell culture inserts (Merck Millipore, Germany) and cultivated for 2 weeks. The medium was refreshed every other day.

### Genome editing

LentiGuide-Puro and lentiCas9-Blast were gifts from Feng Zhang (Addgene plasmids # 52963, http://n2t.net/addgene:52963, RRID:Addgene_52963 and #52962, http://n2t.net/addgene:52962, RRID:Addgene_52962, respectively)^73^. *Streptococcus pyogenes* Cas9 sequence was subcloned into pMXs-Blasti, following which E12 cells were retrovirally transduced. After selection, single guide sequences for C1GALT1 and ST6GALNAC1 were cloned into lentiGuide-Puro, or the non-targetting control. The lentivirus was produced in 293FT cells using the ViraPower Lentiviral Expression System (Thermo Fisher Scientific). The knockout efficiencies were determined by cloning of the genomic sequences corresponding the guide-targeted regions. The targeted regions were PCR amplified from the extracted DNA using following primers: C1GALT1 Forward: 5ʹ-GGGAGAAAAGGTTGACACCCA-3ʹ; C1GALT1 Reverse: 5ʹ-GCGGGTTAATGATGTCACACAG-3ʹ; ST6GALNAC1 Forward-1: 5ʹ-GACAGTGGGTACCTGAACCAG-3ʹ; ST6GALNAC1 Reverse-1: 5ʹ- GACAGTGGGTACCTGAACCAG-3ʹ; ST6GALNAC1 Forward-2: 5ʹ-TCAAGAACGTGCCTCTTGGG-3ʹ; ST6GALNAC1 Reverse-2: 5ʹ-GGGTCTGTGCCTGTGGTTAG-3ʹ; ST6GALNAC1 Forward-3: 5ʹ-TCTGCACAGATTGAGCGGAG-3ʹ; ST6GALNAC1 Reverse-3: 5ʹ-TGTCCAACCCTTTAGAGCCAC-3ʹ. The amplicons were cloned into pCR-Blunt vector (Thermo Fisher Scientific), which was then transformed into DH5α competent cells. A total of 24 colonies (24 colonies for C1GALT1, and 8 colonies per primer pair for ST6GALNAC1) for each knockout cell culture were tested for colony PCR using GoTaq Green Master Mix (Promega, USA). Plasmids from PCR-positive colonies were extracted and subjected to Sanger sequencing.

### Histology and immunofluorescence microscopy

Mouse conjunctiva biopsies were obtained after euthanasia. AB, PAS, or Giemsa staining was performed for PFA-fixed paraffin-embedded sections before and after neuraminidase treatment.

For immunofluorescence microscopy, the biopsy was fixed in 4% PFA at 4 °C for 1 h, washed in PBS, and embedded in O.C.T. compound (Sakura Finetek, Japan). Cryosections were treated with and without neuraminidase (1 U/ml, Nacalai Tesque, Japan) at 37 °C overnight. The specimen was blocked with streptavidin and biotin solutions and Carbo-Free blocking solution (both from Vector Laboratories, USA). Lectin staining was performed with biotinylated Peanut Agglutinin (PNA, Cat.# B-1075-5, Vector Laboratories, 1:1000 dilution), biotinylated Maackia Amurensis Lectin II (MAL-II, Cat.# B-1265-1, Vector Laboratories, 1:200 dilution), or biotinylated Sambucus Nigra Lectin (SNA, Cat.# B-1305-2, Vector Laboratories, 1:1000 dilution) followed by incubation with streptavidin conjugated with AlexaFluor 568 (Cat.# S11226, Thermo Fisher Scientific, 1:1000 dilution), or WGA conjugated with CF488A (Cat.# 29022-1, Biotium, USA). For competition experiments, lectins were incubated with 500 mM of sialic acid (Wako, Japan) prior to application to tissue specimen. Immunostaining was performed with anti-cytokeratin7 (clone EPR17078, Cat.# ab181598, Abcam, UK, 1:1300 dilution), and anti-Sialyl-Tn (clone MLS132, Cat.# 010-25881, FujiFilm, 1:275 dilution) as primary antibodies, and anti-mouse AlexaFluor 568 (Cat.# A11031, ThermoFisher Scientific, 1:200 dilution), anti-mouse AlexaFluor 647 (Cat.# A21236, Thermo Fisher Scientific, 1:200 dilution), anti-rabbit AlexaFluor 488 (Cat.# A11034, Thermo Fisher Scientific, 1:200 dilution), and anti-rabbit IgG Superclonal recombinant antibody AlexaFluor 488 (A27034, Thermo Fisher Scientific, 1:200 dilution) as secondary antibodies. After staining with DAPI (Dojindo, Japan, 1:1000 dilution), the coverslips were mounted with Slowfade Diamond solution (Thermo Fisher Scientific) and fluorescence was observed under the LSM 700 confocal microscope using ZEN 2011 SP3 software (Zeiss, Germany). For separating pollen shell-derived autofluorescence, fluorescence was observed under Nuance Multispectral Imaging System (PerkinElmer).

For the cultured goblet cells, the membranes of culture inserts were rinsed with PBS, fixed in Fixation buffer (Biolegend, USA), and embedded in O.C.T. compound. Sections were stained with either Alcian Blue (pH2.5) and Kernechtrot solution (#4087-1, Muto Pure Chemicals, Japan) or with anti-sialyl-Tn antibody (clone MLS132, FujiFilm, 1:275 dilution) and biotinylated Ulex Europaeus Agglutinin I (UEA-1, Cat.# B-1065-2, Vector Laboratories, 1:500 dilution), followed by anti-mouse AlexaFluor 647 (#A21236, Themo Fisher Scientific, 1:200 dilution) and avidin AlexaFluor 488 (#A21370, Themo Fisher Scientific, 1:200 dilution).

### Enzyme-linked immunosorbent assay (ELISA)

For the detection of sialyl-Tn in the secreted mucin molecule, the conjunctival swab extract was diluted to the concentration of 2 µg/ml and immobilized onto a black microplate at 37 °C for 1 h. The plate was blocked with Carbo-Free blocking solution (SP-5040, Vector Laboratories, USA). After washes, the plates were incubated with 1 µg/ml of sialyl-Tn antibody diluted in PBS (clone MLS132, Cat.# 010-25881, FujiFilm, Japan, 1:1100 dilution) at RT for 30 min. After washes, bound antibody was detected by HRP-conjugated anti-mouse IgG antibody (Cat.# 7076, Cell Signaling Technology, USA, 1:3000 dilution) in PBS containing 1% BSA. After extensive washings, Western Lightning ECL Pro (PerkinElmer) was added as a substrate, and luminescence was measured by GloMax luminescence plate reader (Promega, USA).

For the quantitation of ragweed-specific immunoglobulins in sera, black microplates (Greiner Bio-one, Germany) were coated with 5 μg/mL of RW extract. After blocking with 30% ImmunoBlock reagent (KAC, Japan), diluted mouse serum was applied to the wells and incubated for 2 h at room temperature. After washing, the plates were incubated with biotin-conjugated detection antibodies (rat anti-mouse IgE, clone R35-118, Cat. #553419, BD Biosciences, USA, 1:500 dilution; goat anti-IgG1, #1070-08, SouthernBiotech, USA, 1:10,000 dilution; goat anti-IgG2a, #1080-08, SouthernBiotech, 1:5000 dilution) together with avidin-HRP (Cat.# 405103, BioLegend, CA, USA, 1:1000 dilution) for 1 h. After washings, the plate was subjected to chemiluminescence measurement as described above.

### Flow cytometry

For the measurement of glycan expression, E12 cells were dissociated and fixed with Fixation Buffer (Biolegend, USA) for 20 min at room temperature (RT) before permeabilization with Intracellular Staining Permeabilization Wash Buffer (Biolegend). The permeabilized cells were incubated with anti-sialyl-Tn monoclonal antibody (mAb) (clone MLS132, FujiFilm, Japan, 1:250 dilution) followed by Alexa Fluor 647-conjugated anti-mouse IgG antibody (A21236, Thermo Fisher Scientific, USA, 1:200 diluition).

Conjunctival leukocyte infiltration was evaluated as previously described^69^. Conjunctival tissue was dissected, minced, and digested in RPMI1640 supplemented with 10% FCS, 1 mg/mL collagenase (FujiFilm, Japan) and 0.5 mg/mL DNase I with continuous stirring for 1 h at 37 °C. The dispersed cell suspension was filtered through a nylon mesh. Lymph node cells were directly dispersed using a nylon mesh. The washed cells were blocked with anti-CD16/CD32 antibody (clone 2.4G2, Cat.# 553141, BD Biosciences) for 10 min at 4 °C. The cells were further incubated with surface-staining antibodies at 4 °C for 20 min. For the conjunctival cells, following antibodies were used: anti-CD11b, FITC (clone M1/70, Cat.# 101206, BioLegend, 1:200 dilution), anti-CCR3, PE (clone 83101, Cat.# FAB729P, R&D SYSTEMS, 1:100 dilution), anti-CD45, PerCP/Cyanine5.5 (clone 30-F11, Cat.# 103132, BioLegend, 1:500 dilution), anti-CD11c, PE/Cyanine7 (clone N418, Cat.# 117318, BioLegend, 1:200 dilution), and anti-Siglec-F, Alexa Fluor647 (clone E50-2440, Cat.# 562680, BD Biosciences, 1:500 dilution) for the conjunctival cells. For the lymph node cells, following antibodies were used: Anti-CD11b, FITC (clone M1/70, Cat.# 101206, BioLegend, 1:200 dilution), anti-mouse I-A/I-E, PE (clone M5/114.15.2, Cat.# 107608, BioLegend, 1:1000 dilution), anti-CD45, biotin (clone 30-F11, Cat.# 103104, BioLegend, 1:250 dilution), anti-CD11c, PE/Cyanine7 (clone N418, Cat.# 117318, BioLegend, 1:200 dilution), and streptavidin, BV711 (Cat.# 563262, BD Biosciences, 1:250 dilution). 4ʹ,6-diamidino-2-phenylindole (DAPI, Cat.# 340-07971, Dojindo, 1:1000 dilution) was added to the cell suspension prior to acquisition.

Fluorescence intensity was measured using FACSuite software (BD Biosciences, USA) and FACSVerse flow cytometer (BD Biosciences). Data were analyzed using FlowJo software (BD Biosciences). The gating is shown in Supplementary Fig. 8.

### SDS-PAGE and western blotting

The proteins in the swab extract or the proteins in the secreted mucus were denatured in reducing condition, separated by SDS-PAGE, and electroblotted to PVDF membranes (Millipore). Membranes were stained with AB solution pH 2.5 (Wako, Japan). After differentiation with 3% acetic acid followed by 100% methanol, the membrane was further stained using PAS staining kit (MUTO Pure Chemicals, Japan) according to the manufacturer’s instructions.

For the sialyl-Tn detection by Western blotting, the membranes were blocked with Carbo-Free blocking solution (SP-5040, Vector Laboratories), and incubated with the anti-Sialyl-Tn (clone MLS132, Cat.# 010-25881, FujiFilm, 1:1100 dilution) or anti-Muc5ac (clone 45M1, Cat.# ab3649, Abcam, 1:500 dilution), followed by the HRP-conjugated anti-mouse IgG antibody (Cat.# 7076, Cell Signaling Technology, 1:3000 dilution). After stripping, the same membrane was incubated with WGA-CF488A (Cat.# 29022-1, Biotium, 1:200 diliution). The ECL luminescence and the fluorescence were visualized with LAS-4000 (FujiFilm).

### Micorarray analysis

Total RNA was extracted from mouse conjunctival tissues using RNeasy Mini Kit (Qiagen, Germany) according to the manufacturer’s instructions. The same amount of total RNA from 3 mice were mixed for each cohort and a microarray analysis was performed using Clariom S assay for mouse (Thermo Fisher Scientific, USA) following the manufacturer’s instruction. Data were analyzed by Transcriptome Analysis Console Software (Thermo Fisher Scientific).

### Cloning and retroviral transduction of St6galnac1

*St6galnac1* transcripts were amplified from the mouse conjunctival cDNA by PCR using Q5 DNA polymerase (New England Biolabs, USA). The amplicons were cloned into pCR-Blunt vector (Thermo Fisher Scientific), which was then transformed into DH5α competent cells. Colony PCR was performed using GoTaq Green Master Mix (Promega, USA). The obtained clones were sequenced and subcloned into pMXs-IG vectors. Retrovirus was produced by transfection into Plat-A cells, and then applied to E12 cells.

### Sanger sequencing

The genomic DNA was extracted from mouse tails and the *St6galnac1* genomic sequence containing exon 5 (PCR region 2 in Supplementary Fig. 3b) was amplified with B6J primers (Supplementary Table 1). The amplicon was purified and Sanger-sequenced using Common-R primer for region 2 in the laboratory of FASMAC (FASMAC, Japan).

### Collection of secreted mucus

The shells of RW pollen were produced as previously described^69^. The RW pollens were defatted with acetone at 60 °C overnight, followed by an incubation in phosphoric acid at 60 °C for 7 d. In this step, most of the pollen content was removed from the navel pore. After washings, the pollens were further incubated in 6% w/v KOH solution at 80 °C for 6 h. The process was repeated to remove heat- and base-labile components such as proteins and endotoxins. The washed pollen shells were dried overnight. The shells were weighed and suspended in PBS at the same particle concentrations as 200 mg/mL of RW pollen suspension.

Each mouse conjunctival sac received 2.5 μL of shell suspension to induce mucus secretion. Twenty-five min later, the mice were deeply anesthetized or sacrificed, and the pollen shell-mucus aggregates were carefully collected from the conjunctival sac. The morphology of the aggregates was observed under a stereo microscope. The aggregates were either fixed in 4% PFA overnight at 4 °C before immunostaining, treated with or without neuraminidase at 37 °C for two days before Western Blotting, directly subjected to reverse penetrability assessment, or subjected to the measurement of protein concentration.

### Measurement of protein concentration and pollen shell amount

The protein amount was measured using Micro BCA Protein Assay Kit (ThermoFisher) following manufacturer’s instructions. After measurement, the released pollen shells were washed in water, and their amount was measured using their autofluorescence at an excitation wavelength 405 nm and an emission filter 500–550 nm using GloMax luminescence plate reader (Promega).

### Reverse penetration assay

The pollen aggregates collected in PBS was soaked in PBS containing FluoSphere Carboxylate-Modified Microspheres, 0.5 µm, red fluorescent (580/605) (#F8812, ThermoFisher) and/or WGA-CF488A (Biotium) in a well of µ-Plate 96 Well Black plate (#89626, ibidi, Germany). The fluorescence was observed under the LSM 700 confocal microscope (Zeiss) using ZEN2011 SP3 software (Zeiss).

### Human samples

Six control subjects and seven atopic keratoconjunctivitis patients were recruited. Clinical parameters other than diagnosis, such as age and sex information were not collected. The recruitment was announced for consecutive patients who were visiting the Department of Ophthalmology Juntendo University, Juntendo and Urayasu Hospital, Tokyo and Chiba, Japan. When conjunctival samples were collected from AKC patients, there was no treatment of eye drops to these patients. Healthy volunteers who did not show signs of allergic conjunctivitis or corneal diseases were recruited. Written informed consent was received prior to participation. Impression cytology specimens were obtained using EYEPRIM (OPIA Technologies SAS, France) as shown in Fig. 1a. The cells were fixed with 4% paraformaldehyde in PBS, rinsed with PBS, and embedded in O.C.T. compound. The sections were subjected to AB and PAS staining, HID staining, or immunostaining. In some cases, sections were treated with neuraminidase from Arthrobacter ureafaciens (#24229-74, Nacalai Tesque, Japan) before staining.

### Statistics & reproducibility

Statistical analysis was performed using Prism 8 software (GraphPad, CA). Data are presented as mean ± S.E.M. in all figure parts in which error bars are shown. No statistical method was used to predetermine sample size. We determined sample sizes by referring to previously published papers in the field^56,69^. The investigators were blinded to allocation during experiments and outcome assessment. Groups were analyzed by two-tailed tests. *P* values of <0.05 were considered statistically significant. All experiments except for the one using human samples were replicated at least twice. All attempts to replication were successful. Human experiment (Fig. 7b) was not replicated because of the limitation of the sample availability. Analysis based on the published database (Fig. 7c) was not replicated because of the data availability.

### Reporting summary

Further information on research design is available in the Nature Portfolio Reporting Summary linked to this article.

## Supplementary information

Supplementary Information

Reporting Summary

## Data Availability

Source data are provided with this paper. The microarray data for mouse conjunctival gene expression profiles is deposited in Gene Expression Omnibus (GEO; accession no. GSE220182). The genomic DNA sequences for various mouse strains were retrieved through Ensembl database (https://www.ensembl.org). Publicly available dataset GSE155776 was retrieved from Gene Expression Omnibus. Source data are provided with this paper.

## Source data

Source Data
