# Supplementary material for: The protective role of conjunctival goblet cell mucin sialylation
Source: Nat Commun. 2023 Mar 17;14:1417. doi: 10.1038/s41467-023-37101-y (PMC10023771; doi:10.1038/s41467-023-37101-y)

Supplementary Information

The protective role of conjunctival goblet cell mucin sialylation

Moe Matsuzawa, Tomoaki Ando, Saaya Fukase, Meiko Kimura, Yasuharu Kume, Takuma Ide, Kumi Izawa, Ayako Kaitani, Mutsuko Hara, Eri Nakamura, Anna Kamei, Akira Matsuda, Nobuhiro Nakano, Keiko Maeda, Norihiro Tada, Hideoki Ogawa, Ko Okumura, Akira Murakami, Nobuyuki Ebihara, and Jiro Kitaura

Content:

Supplementary Tables 1-2

Supplementary Figures 1-11

Supplementary Source Data

Supplementary Table 1. Oligos used for this study.

**qPCR primers**		
Muc1	F	CAACCCAGGACACCTACCATC
	R	TTACCTGCCGAAACCTCCTCA
Muc4	F	ACCAGATGGCTCTGAACCTAAGTA
	R	GGTGGTAGCCTTTGTAGCCAT
Muc16	F	CGGATCAGTGGAAGGCATGA
	R	GCTTGTTCCATGGTTGGCAG
Muc5ac	F	CTTCTGTCCTGAGGGTATGGTG
	R	CATGTGTTGGTGCAGTCAGTAGA
St6galnac1	Fq	AGCCTCGGTGGGATTTTGAG
	Rq	CAGAAGCAGAGCAGTTCGTC
	F3	CTGTGCTGTAGTGGGCAACG
	R3	GGCTCCACTCAGTCGGAAA
	F4	TGACTATGTTTTCCGACTGAGTG
	R4	CATGCTGGAAGCCTCGTCTA
St6galnac2	F	GGGACAGGCCTCACACCTATT
	R	TGATGAAGTCTGGGTGCAGG
C1galt1	F	TGGGAAAGAAACCTTCCATCCAT
	R	TCCAGGACCCTCTATGGGAG
C1galt1c1	F	GGAGCCGTTCTAGATGCGGAAA
	R	GATGCTCATGGTGGTGCATTC
St3gal4	F	CTGCGCGCGGATTTACCT
	R	CAGGAGCTTCCAGTGAGATTTGCT
St6gal1	F	ACCATCCGCCTAGTGAACTC
	R	ACACAGATGGGTCCCACAGA
St8sia6	F	CCAGTGTGCAGTGGTTGGTA
	R	ACTAGCGCTCCCTGTGATTG
Gcnt3	F	ACTCACGGCGTTGGAGAAAG
	R	CACAGGCGAGGACCATCAAG
Neu1	F	TAAGGACGACGGCATTTCCT
	R	GTCCCGTGTCCACACACAAT
**Cloning primers**		
St6galnac1	Cloning F	AAA CTCGAG CTGTTAGGGACCAGCCATCC
	Cloning R	AAA GCGGCCGC GAGGAGCCCTTGCATTCTGT
Colony PCR	F1	CAGGAAACAGCTATGAC
	R1	GTAAAACGACGGCCAG
St6galnac1 Nested PCR	F2	CCTGTGCTGTAGTGGGCAAC
	R2	CTGGAAGCCTCGTCTACCC
**Genotyping PCR**		
Region 1	B6J-F	GTATGTTCTCCCTCCGTTGCT
Region 1	B6J-R	TGTGGAGATGAATCAGGAGGC
Region 2/4	B6J-F	TGTGTCTACAGCTCCTGCCT
Region 2/4	Balb/Gm11735-F	AGTGTGTCTACAGCTCCTACCC
Region 2	Common-R	CTCATTGGTCTCCCATGTTCAGG
Region 4	B6J/Balb-R	TTTCCATCCACATACAAGGCG
Region 3	B6J-F	TCTTTCTACACAG
Region 3	Common-R	TTCTCACCTTTC
**sgRNA preparation**		
ST6GALNAC1	F	CACCGAAGGCACCCGGGACTATGAG
	R	AAACCTCATAGTCCCGGGTGCCTTC
C1GALT1	F	CACCGTATTGCTGATCCACAGAGGA
	R	AAACTCCTCTGTGGATCAGCAATAC
Non-targeting control 1	F	CACCGACTCGACCTAACGTCGATGT
	R	AAACACATCGACGTTAGGTCGAGTC
Non-targeting control 2	F	CACCGTGTGATCCACCGATCGTAT
	R	AAACATACGATCGGTGGATCACAC

Supplemental Table 2. Comparisons of sialyltransferase amino acid sequences between B6J and Balb/c strains.

Sialyltransferase	Ensembl canonical mRNA	peptide length (a.a.)	Balb/c mRNA	peptide length (a.a.)	Comparision of peptide sequences
St6galnac1	ENSMUST00000009732.8	526	MGP_BALBcJ_T0033675.1	553	This study found new transcripts in Balb/c (Figure 2f)
St6galnac2	ENSMUST00000079545.6	373	MGP_BALBcJ_T0033669.1	373	Identical
St6galnac3	ENSMUST00000200397.5	305	MGP_BALBcJ_T0064346.1	305	Identical
St6galnac4	ENSMUST00000102818.11	302	MGP_BALBcJ_T0054114.1	302	Identical
St6galnac5	ENSMUST00000044278.6	335	MGP_BALBcJ_T0064344.1	336	Q48dup in Balb/c
St6galnac6	ENSMUST00000081879.12	335	MGP_BALBcJ_T0054125.1	335	Identical
St6gal1	ENSMUST00000023601.14	403	MGP_BALBcJ_T0043849.1	403	Identical
St6gal2	ENSMUST00000025000.4	524	MGP_BALBcJ_T0048155.1	524	R48S in Balb/c
St3gal1	ENSMUST00000229028.2	337	MGP_BALBcJ_T0041093.1	337	Identical
St3gal2	ENSMUST00000034197.5	350	MGP_BALBcJ_T0090478.1	350	Identical
St3gal3	ENSMUST00000030263.9	374	MGP_BALBcJ_T0067342.1	374	Identical
St3gal4	ENSMUST00000034537.8	333	MGP_BALBcJ_T0091643.1	333	E65D in Balb/c
St3gal5	ENSMUST00000069994.11	414	MGP_BALBcJ_T0078051.1	414	Identical
St3gal6	ENSMUST00000137035.8	329	MGP_BALBcJ_T0044700.1	329	Identical
St8sia1	ENSMUST00000032421.4	355	MGP_BALBcJ_T0080903.1	355	Identical
St8sia2	ENSMUST00000026896.10	375	MGP_BALBcJ_T0084463.1	375	Identical
St8sia3	ENSMUST00000025477.15	380	MGP_BALBcJ_T0049816.1	380	Identical
St8sia4	ENSMUST00000043336.11	359	MGP_BALBcJ_T0021469.1	359	Identical
St8sia5	ENSMUST00000075290.8	412	MGP_BALBcJ_T0050076.1	412	Identical
St8sia6	ENSMUST00000003509.10	398	MGP_BALBcJ_T0052609.1	398	Identical

Supplementary Figure 1

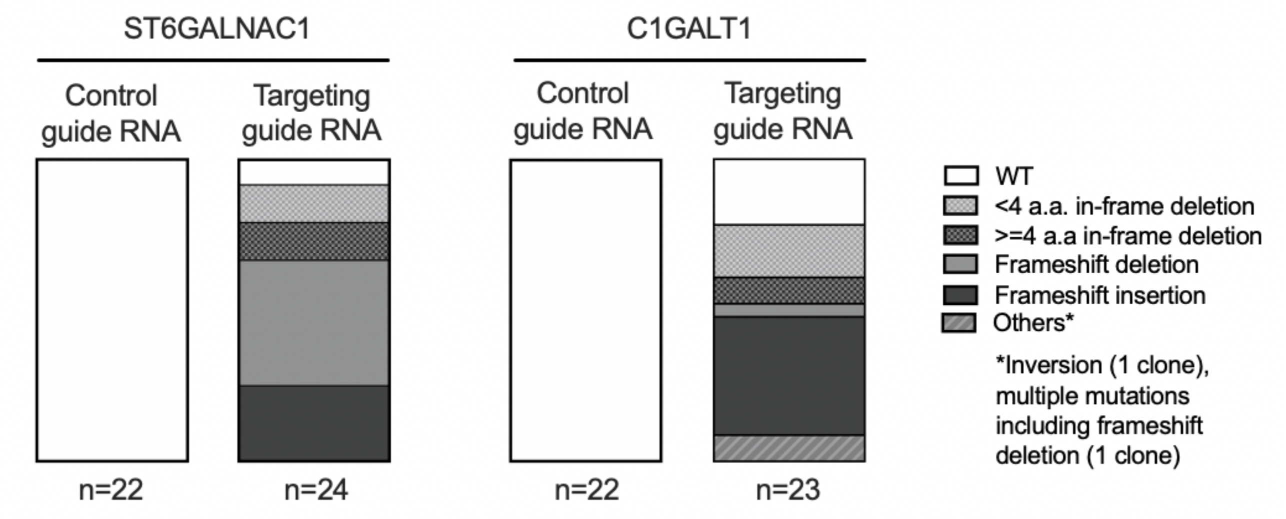

Supplementary Fig. 1. Genome editing results of ST6GALNAC1 and C1GALT1.

The mutation patterns were determined by Sanger sequencing of indicated target regions. Source data are provided as a Source Data file.

Supplementary Figure 2

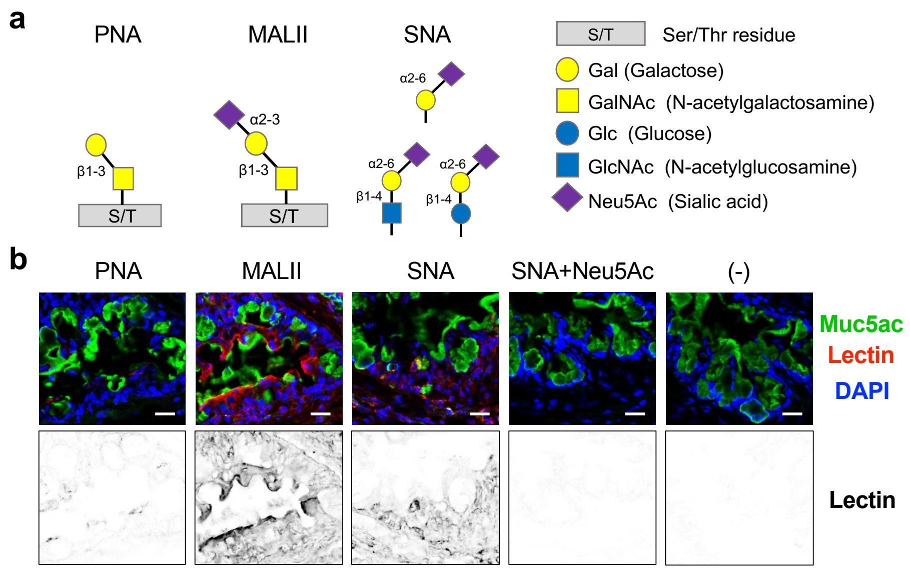

Supplementary Fig. 2. Lectins detected no sialic acid linked to galactose residue in conjunctival goblet cells.

a Specificities of lectins used. **b** B6J conjunctiva was stained with the indicated lectins together with anti-Muc5ac antibody. Data are representative of two independent experiments. Bar, 20 μm.

Supplementary Figure 3

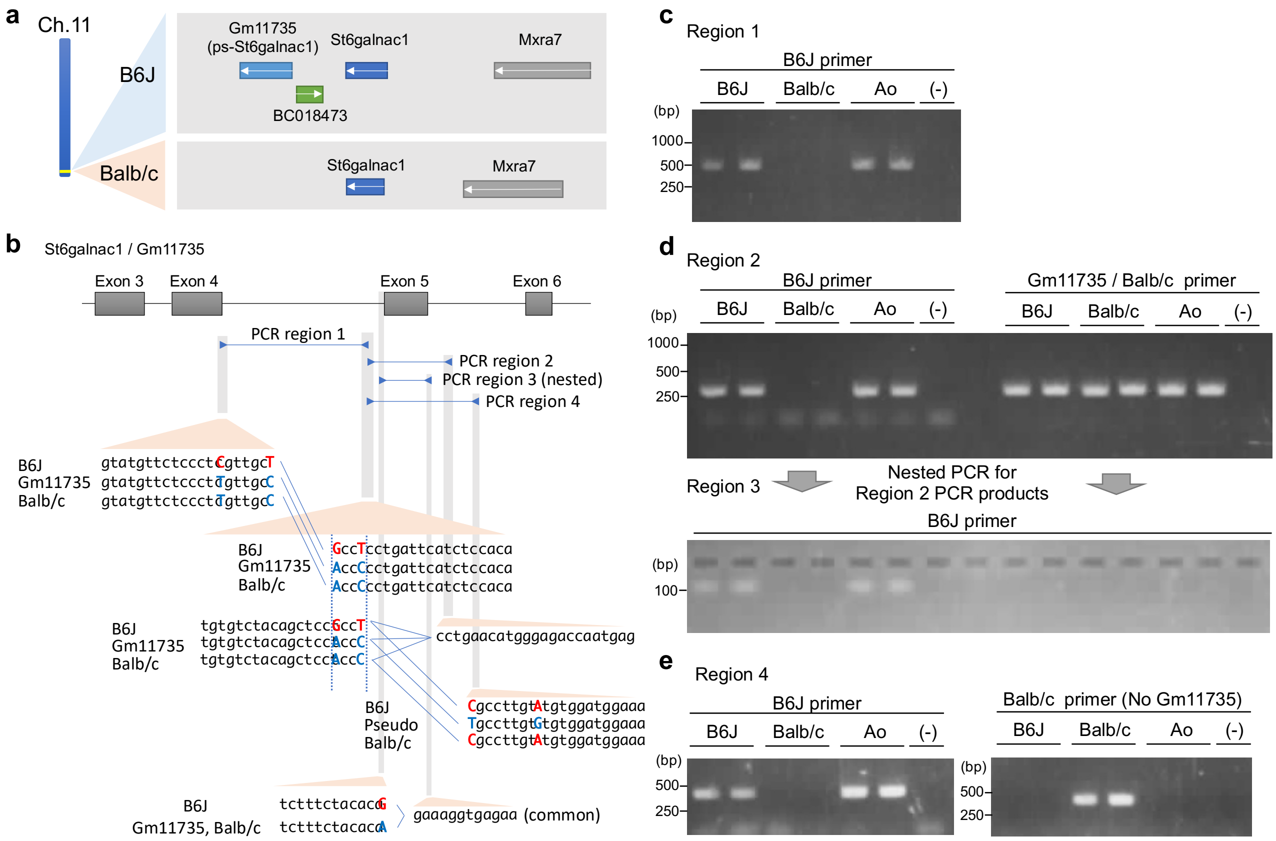

Supplementary Fig. 3. Genotyping strategy for B6J-derived intact St6galnac1 allele.
a Gene coordination of B6J and Balb/c strains near St6galnac1 gene on chromosome (Ch.) 11. B6J has an extra pseudogene for St6galnac1 (Gm11735). **b** Genotyping primers and genomic sequences of St6galnac1 and Gm11735 pseudogene. Nested PCR (region 3) can distinguish the single base mutation at the 3' splice site before exon 5. **c-e** Genotyping PCR results of B6J, Balb/c, and Ao strains for regions 1 (**c**), 2 and 3 (**d**), and 4 (**e**). The PCR results for two mice for each strain are shown. Source data are provided at the end of Supplementary Information.

Supplementary Figure 4

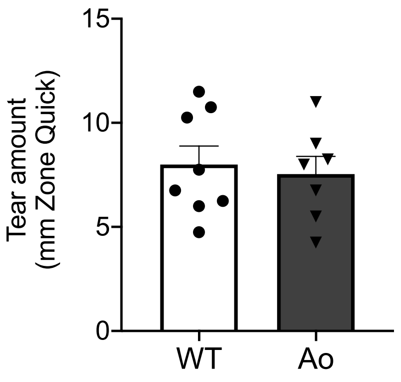

Supplementary Fig. 4. Tear volume was unaffected by the presence of sialic acid. The tear amounts of steady state WT Balb/c (n=8) and Ao (n=7) mice were measured by Zone-quick phenol red threads and shown as an averaged wet lengths for both eyes. Data are representative of at least two independent experiments. Source data are provided as a Source Data file.

Supplementary Figure 5

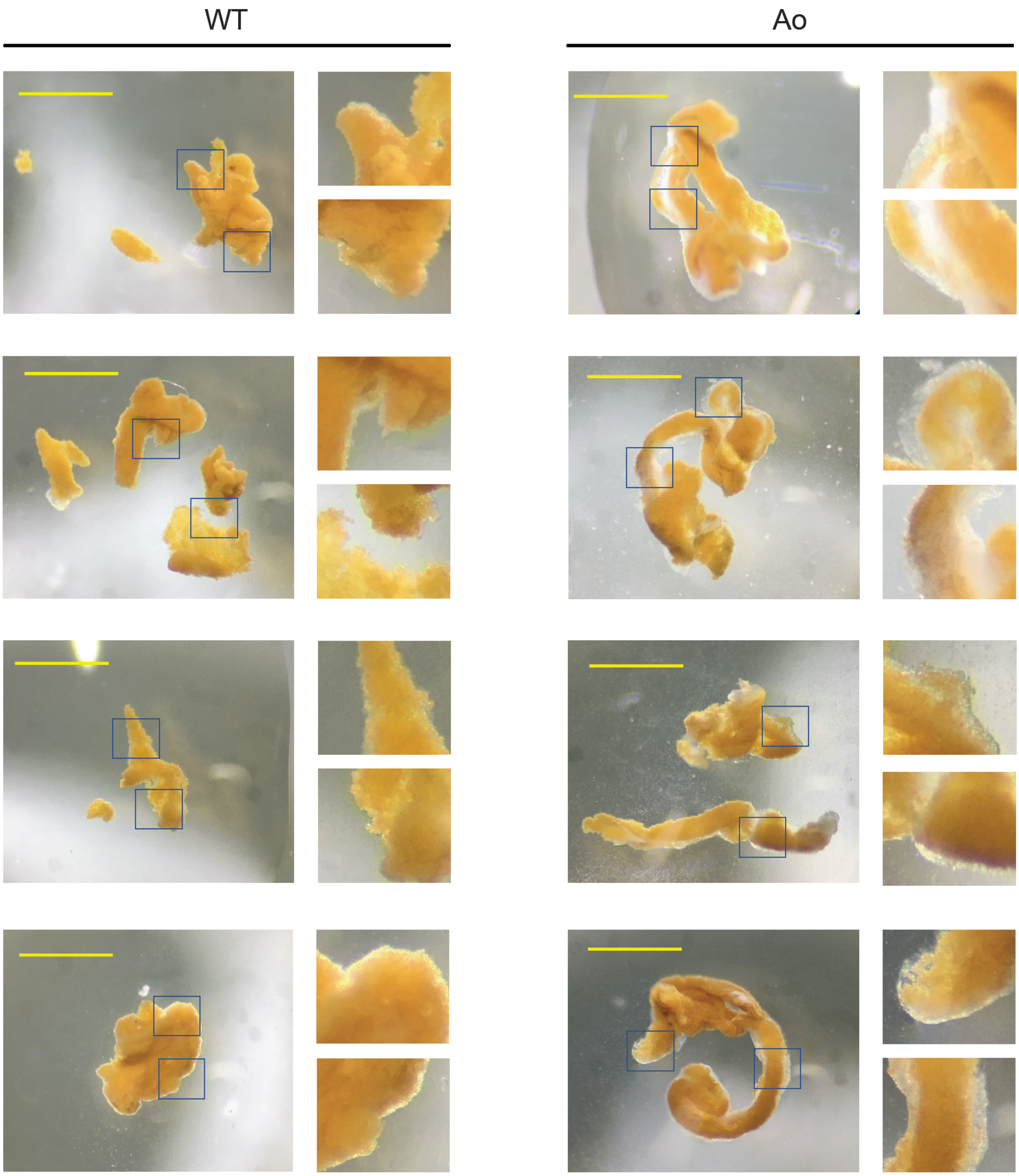

Supplementary Fig. 5. Pollen shells are encapsulated by mucus layer in Ao mice. More representative pictures related to Fig. 5. Bar, 1 mm. Data are representative of at least three independent experiments.

Supplementary Figure 6

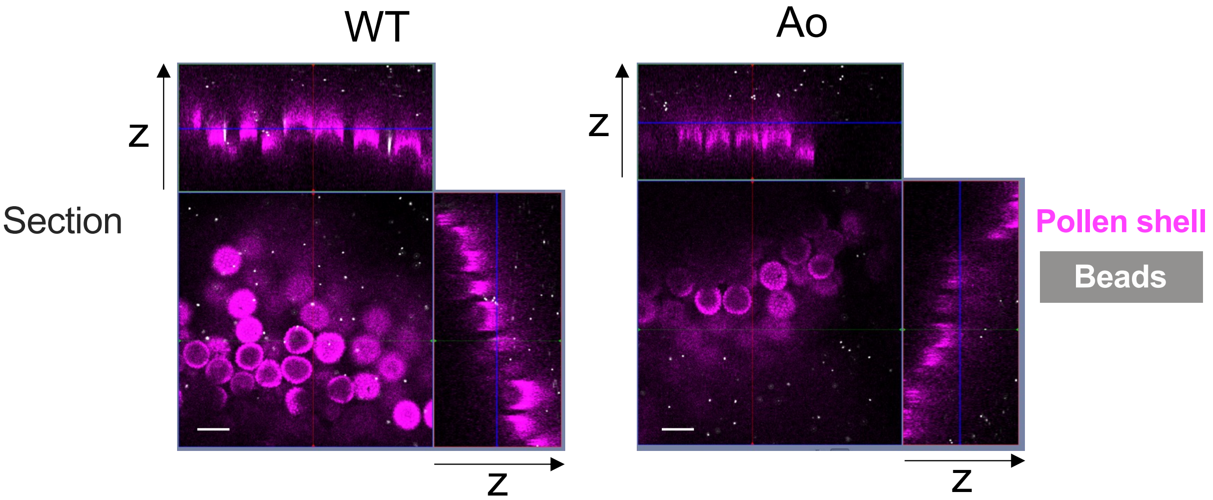

Supplementary Fig. 6. Fluorescent beads have access to the pollen shell surface of the aggregate from WT, but not to that of the aggregate from Ao mice.

The pollen aggregates shown in Fig 5b were shown in X-Y, X-Z, and Y-Z sections. Data are representative of two independent experiments. Bar, 20 μm.

Supplementary Figure 7

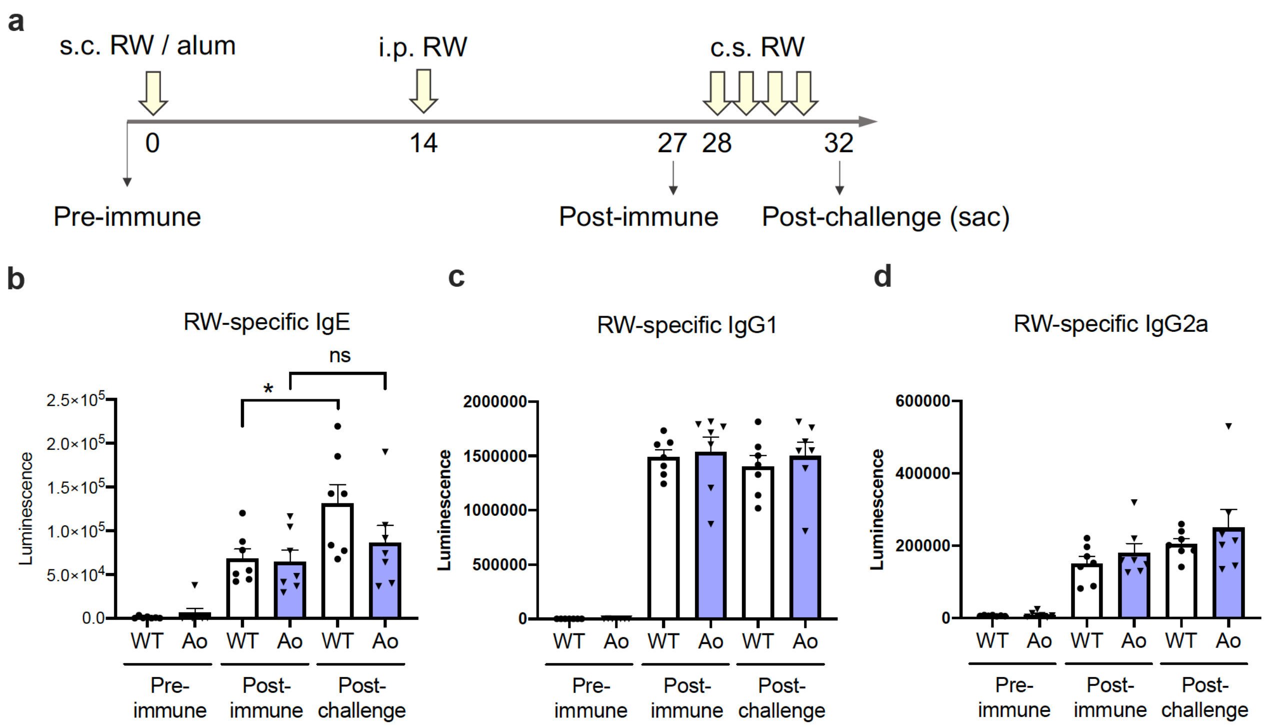

Supplementary Fig. 7. Humoral responses were not affected at the sensitization phase. **a** Schematic diagram of the allergic conjunctivitis model. Timing of blood drawing is shown in thin arrows. s.c., subcutaneous injection; i.p., intraperitoneal injection; c.s., instillation into conjunctival sac; sac, sacrifice. **b-d** Levels of RW-specific IgE (**b**), IgG1 (**c**), and IgG2a (**d**). n=7 for each strain. *p<0.05 (adjusted p = 0.0142) and ns, not significant (adjusted p = 0.0796) by two-tailed matched ANOVA with Geisser-Greenhouse correction and Holm-Sidak multiple comparisons for indicated pairs. Data are representative of at least three independent experiments. Source data are provided as a Source Data file.

Supplementary Fig 8.

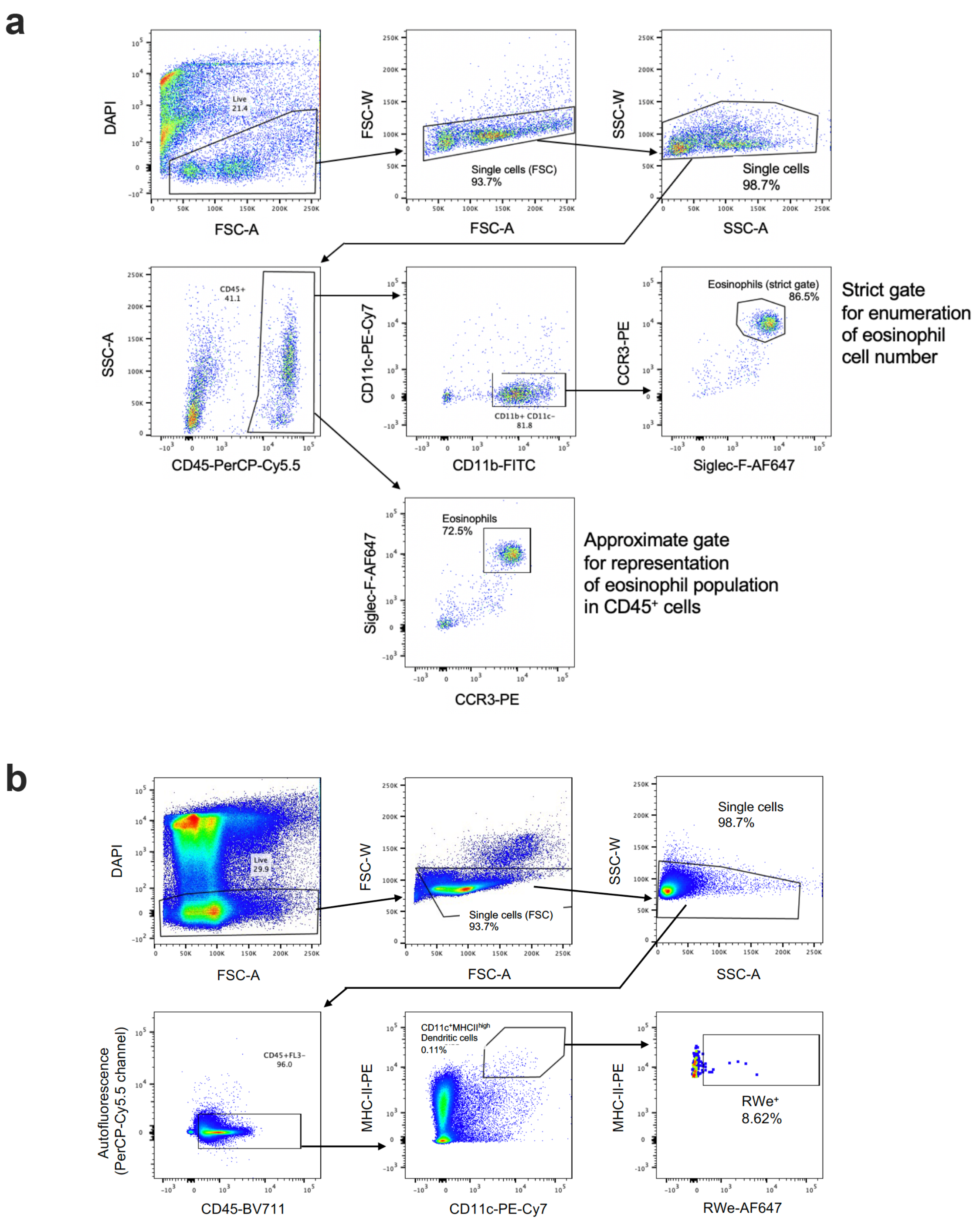

Supplementary Fig. 8 The gating strategy for eosinophils in the conjunctiva and migratory dendritic cells in the lymph nodes.

a A gating example for the conjunctival eosinophils. A representative sample from allergic conjunctivitis-induced wild-type Balb/c mouse is shown. **b** A gating example for the migratory dendritic cells. A representative sample from RW extract (RWe) labeled with AF647-instilled wild-type Balb/c mouse is shown.

Supplementary Figure 9

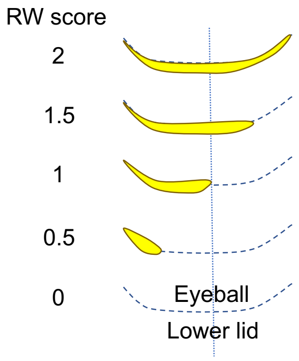

Supplementary Fig. 9 Scoring of RW pollens left in the conjunctiva.

RW pollens applied to the lower conjunctival sac distribute along the edge of the eyeball and the lower lid. The amount of RW pollens left in the sac is scored by the total length of the pollen aggregate.

Supplementary Figure 10

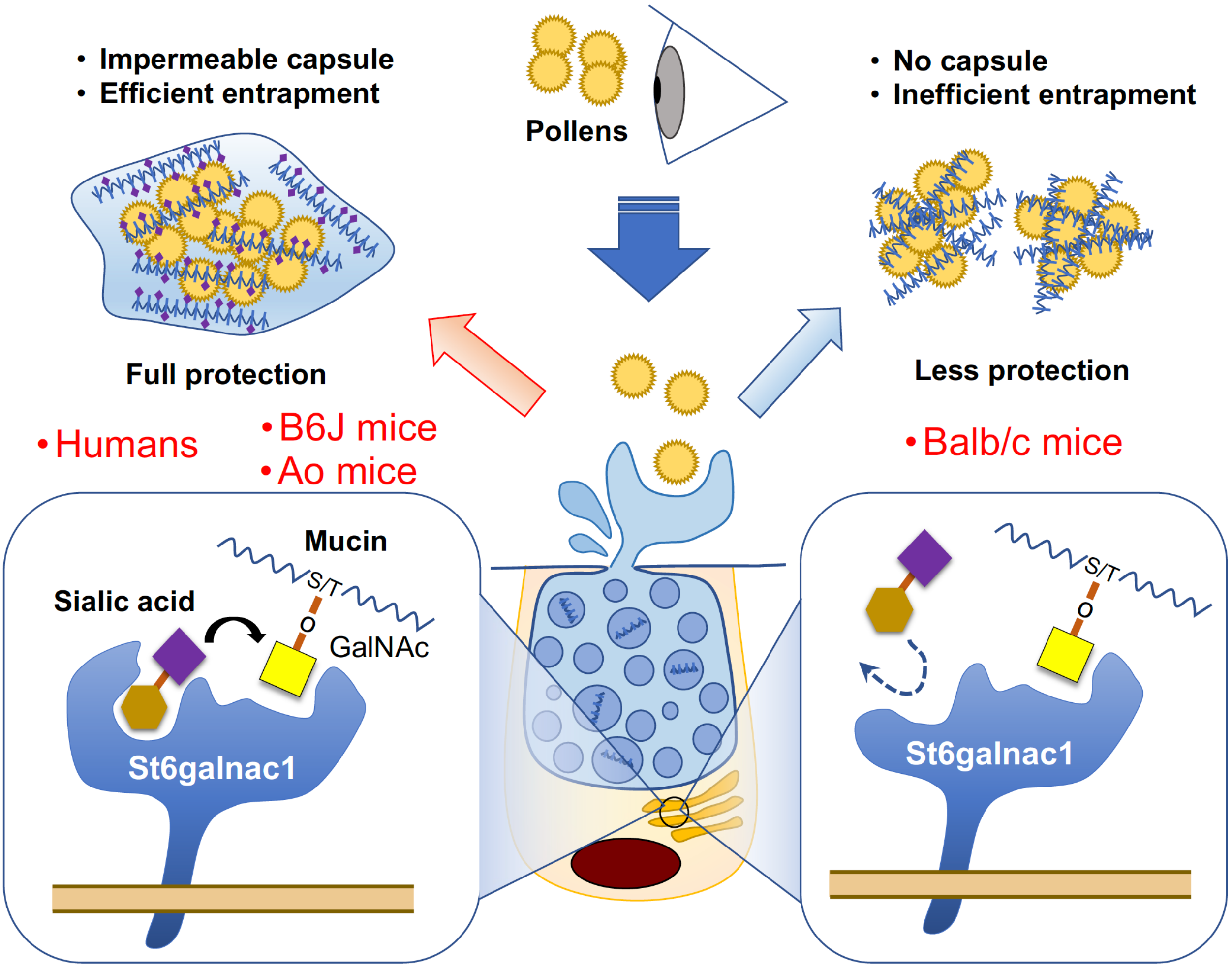

Supplementary Fig. 10. Schematic summary of the findings. Upon detection of the pollen particles, the conjunctival goblet cells secrete mucus containing Muc5ac. The mucus is AB-positive due to sialylation in humans and B6J mice, but not in Balb/c mice. Balb/c mice have a point mutation in a sialyltransferase *St6galnac1*. The sialylation was required for the efficient entrapment and encapsulation of the pollen particles with an impenetrable layer. Ao mice, which are fully Balb/c-backcrossed mice with intact *St6galnac1* alleles, are protected against allergic conjunctivitis. Human conjunctival goblet cells express ST6GALNAC1 and/or sialyl-Tn in conditions under chronic stimuli.

Supplementary Figure 11

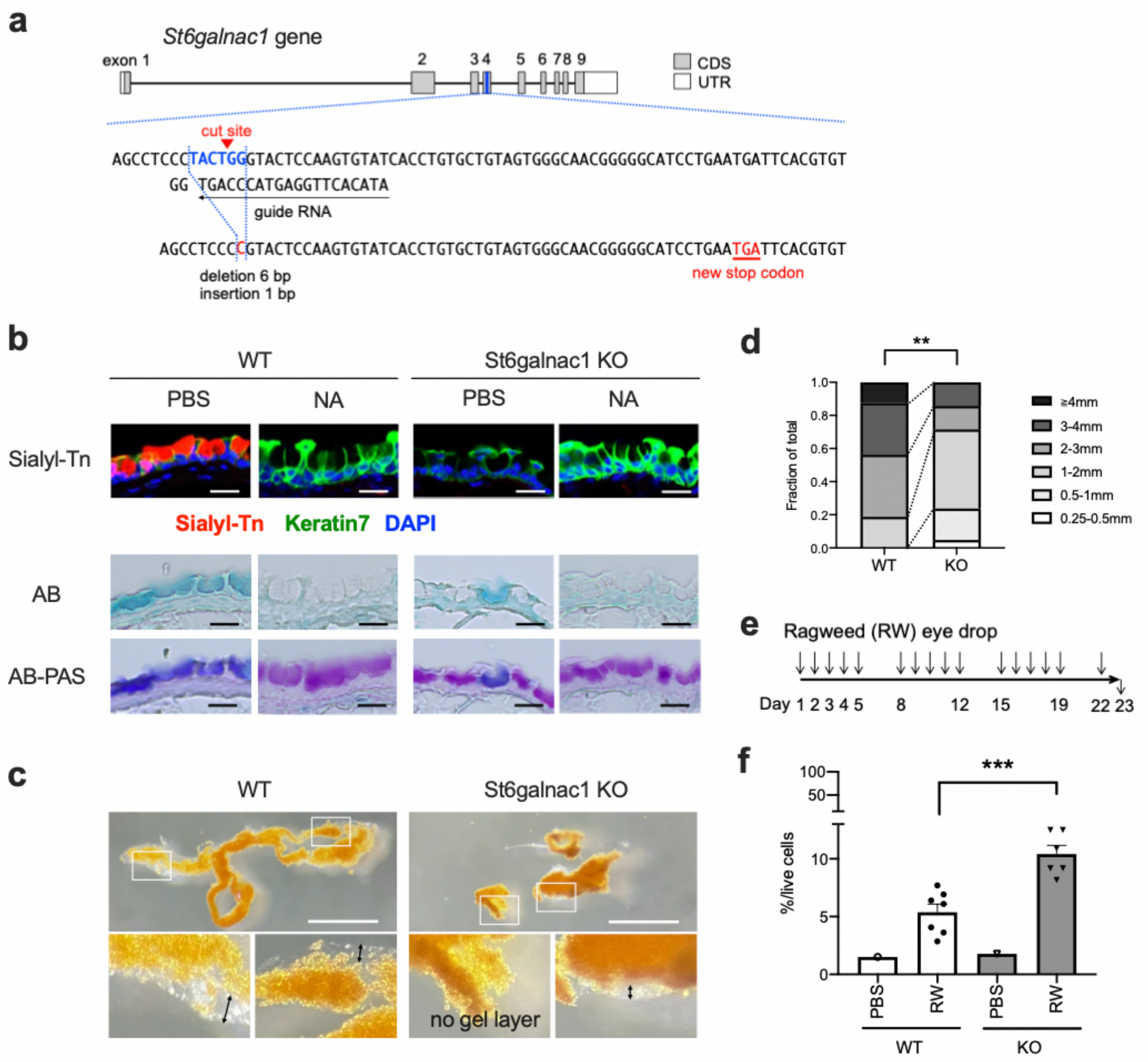

Supplementary Fig. 11. St6galnac1 knockout mice on B6J background present less-protective phenotype against allergic conjunctivitis compared to wild type mice.
a Schematic representation of *St6galnac1* genome editing for *St6galnac1* knockout (KO) mouse generation. **b** PFA-fixed frozen sections were immunostained for sialyl-Tn followed by Alcian blue (AB) and Periodic Acid-Schiff (PAS) staining. Bar, 20 µm. **c,d** The representative image (**c**) and length distribution (**d**) of pollen aggregates retrieved from WT (n=7) and KO (n=6) mice instilled with ragweed pollen suspension. Arrows indicate mucus layer thickness. Bar, 1 mm. ***p*<0.01 (p = 0.0025) by two-tailed Fisher's exact test for the ratio of fractions <2 mm and ≧2 mm. Data are representative of at least two independent experiments. **e** An experimental schedule for the induction of the allergic conjunctivitis model. **f** Eosinophil frequencies in live single cells. n=1, 7, 1, and 6 for WT-PBS, WT-RW, KO-PBS and KO-RW, respectively. ****p*<0.001 (p = 0.0005) by two-tailed Welch's t-test. Data are representative of at least three independent experiments. Source data are provided as a Source Data file.

Supplementary Source Data

Supplemental Fig 3c

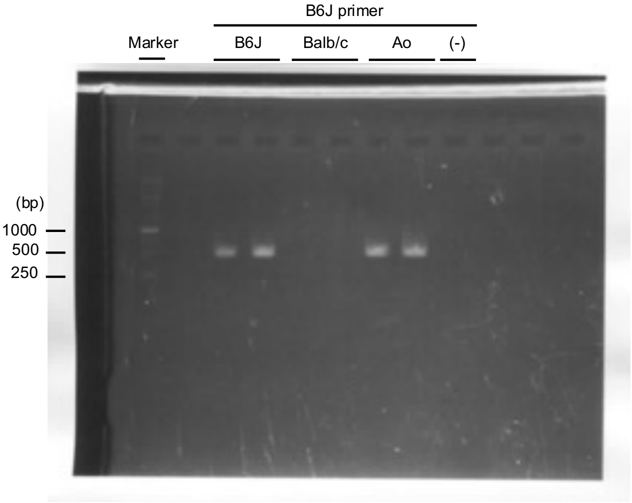

Supplementary Fig 3d

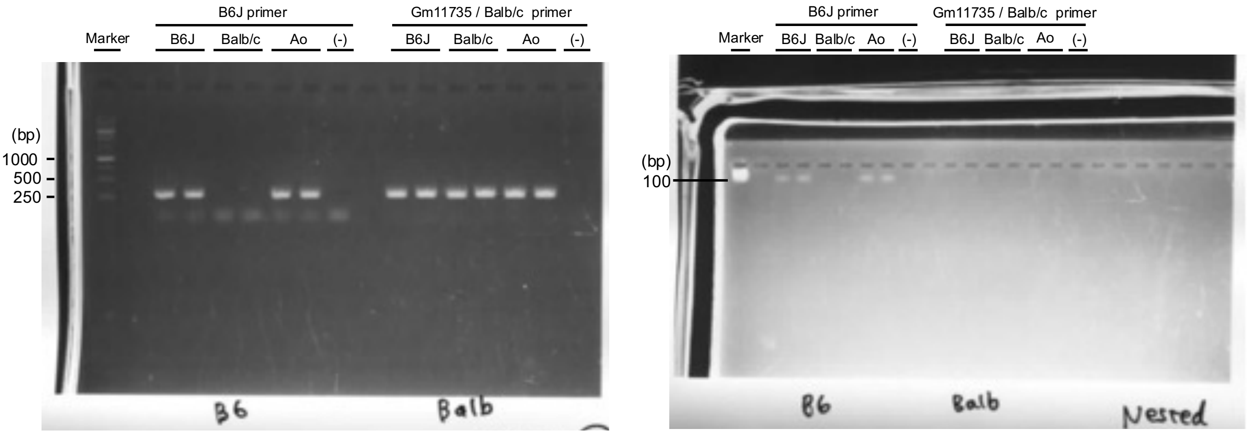

Supplementary Fig 3e

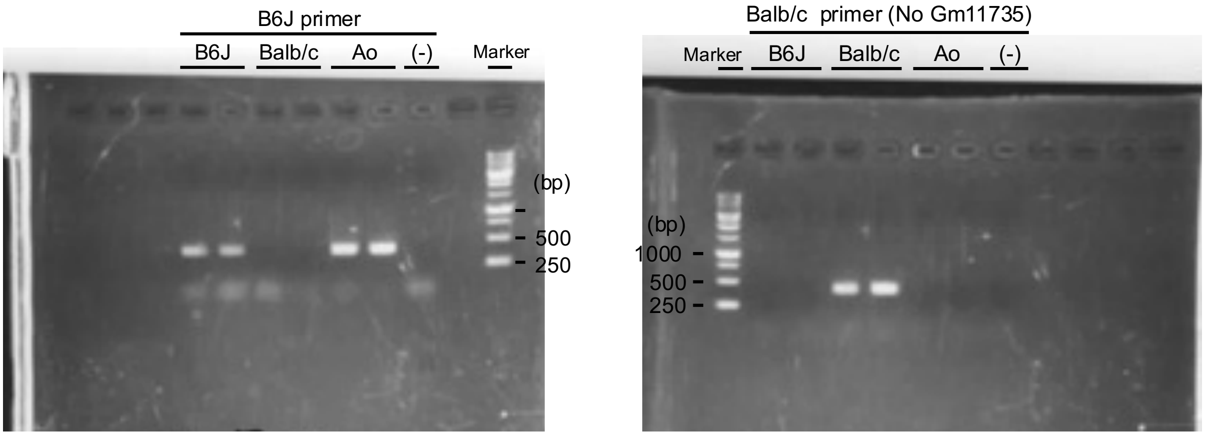

Supplement: Supplementary file 1 — Supplementary Information [file 41467_2023_37101_MOESM1_ESM.pdf]
