# Supplementary material for: The protective role of conjunctival goblet cell mucin sialylation
Source: Nat Commun. 2023 Mar 17;14:1417. doi: 10.1038/s41467-023-37101-y (PMC10023771; doi:10.1038/s41467-023-37101-y)
Supplement: Supplementary file 2 — Reporting Summary [file 41467_2023_37101_MOESM2_ESM.pdf]

nature portfolio

Corresponding author(s): Tomoaki Ando

Last updated by author(s): Jan 27, 2023

Reporting Summary

Nature Portfolio wishes to improve the reproducibility of the work that we publish. This form provides structure for consistency and transparency in reporting. For further information on Nature Portfolio policies, see our Editorial Policies and the Editorial Policy Checklist.

Statistics

For all statistical analyses, confirm that the following items are present in the figure legend, table legend, main text, or Methods section.

n/a	Confirmed	
☐	☒	The exact sample size (*n*) for each experimental group/condition, given as a discrete number and unit of measurement
☐	☒	A statement on whether measurements were taken from distinct samples or whether the same sample was measured repeatedly
☐	☒	The statistical test(s) used AND whether they are one- or two-sided<br>*Only common tests should be described solely by name; describe more complex techniques in the Methods section.*
☒	☐	A description of all covariates tested
☐	☒	A description of any assumptions or corrections, such as tests of normality and adjustment for multiple comparisons
☐	☒	A full description of the statistical parameters including central tendency (e.g. means) or other basic estimates (e.g. regression coefficient) AND variation (e.g. standard deviation) or associated estimates of uncertainty (e.g. confidence intervals)
☐	☒	For null hypothesis testing, the test statistic (e.g. *F*, *t*, *r*) with confidence intervals, effect sizes, degrees of freedom and *P* value noted<br>*Give P values as exact values whenever suitable.*
☒	☐	For Bayesian analysis, information on the choice of priors and Markov chain Monte Carlo settings
☒	☐	For hierarchical and complex designs, identification of the appropriate level for tests and full reporting of outcomes
☒	☐	Estimates of effect sizes (e.g. Cohen's *d*, Pearson's *r*), indicating how they were calculated

Our web collection on statistics for biologists contains articles on many of the points above.

Software and code

Policy information about availability of computer code

Data collection	Flow cytometry: BD FACSuite (1.0.6)<br>Confocal microscopy: ZEN 2011 SP3 (Black Edition)
Data analysis	Statistics: GraphPad Prism 8 (8.4.3)<br>Flow cytometry: Flowjo (10.8.1)<br>Transcriptome Analysis Console (4.0.2)

For manuscripts utilizing custom algorithms or software that are central to the research but not yet described in published literature, software must be made available to editors and reviewers. We strongly encourage code deposition in a community repository (e.g. GitHub). See the Nature Portfolio guidelines for submitting code & software for further information.

Data

Policy information about availability of data

All manuscripts must include a data availability statement. This statement should provide the following information, where applicable:

- Accession codes, unique identifiers, or web links for publicly available datasets
- A description of any restrictions on data availability
- For clinical datasets or third party data, please ensure that the statement adheres to our policy

The microarray data for mouse conjunctival gene expression profiles is deposited in Gene Expression Omnibus (GEO; accession no. GSE220182; https://

www.ncbi.nlm.nih.gov/geo/query/acc.cgi?acc=GSE220182).
The genomic DNA sequences for various mouse strains were obtained through Ensembl database (https://www.ensembl.org).
Publicly available dataset GSE155776 was retrieved from Gene Expression Omnibus (https://www.ncbi.nlm.nih.gov/geo/query/acc.cgi?acc=GSE155776).

Human research participants

Policy information about studies involving human research participants and Sex and Gender in Research.

Reporting on sex and gender	Sex and gender information has not been collected.
Population characteristics	6 control subjects and 7 atopic keratoconjunctivitis patients were recruited. Clinical parameters other than diagnosis, such as age and sex information was not collected.
Recruitment	The recruitment was announced for consecutive patients who were visiting the Department of Ophthalmology Juntendo University, Juntendo and Urayasu Hospital, Tokyo and Chiba, Japan. When conjunctival samples were collected from AKC patients, there was no treatment of eye drops to these patients. Healthy volunteers who did not show signs of allergic conjunctivitis or corneal diseases were also recruited. Written informed consent was received prior to participation. There could be a referral bias because this study was conducted in advanced medical institutions.
Ethics oversight	All human procedures were approved by the Ethical Review Board, Juntendo University Faculty of Medicine (approval no. 2019244). The study was conducted in accordance with the tenets of the Declaration of Helsinki.

Note that full information on the approval of the study protocol must also be provided in the manuscript.

Field-specific reporting

Please select the one below that is the best fit for your research. If you are not sure, read the appropriate sections before making your selection.

☒ Life sciences ☐ Behavioural & social sciences ☐ Ecological, evolutionary & environmental sciences

For a reference copy of the document with all sections, see nature.com/documents/nr-reporting-summary-flat.pdf

Life sciences study design

All studies must disclose on these points even when the disclosure is negative.

Sample size	No statistical methods were used to predetermine sample sizes. We determined sample sizes by referring to previously published papers in the field such as Fukase et al, Ocul Surf. 2021 (PMID: 34428578). Basically, at least triplicate experiments were performed to meet the minimal requirements for statistical analysis.
Data exclusions	No data were excluded from the analyses.
Replication	All experiments except for the one using human samples were replicated at least twice. All attempts to replication were successful. Human experiment (Figure 7b) was not replicated because of the limitation of the sample availability. Analysis based on the published database (Figure 7c) was not replicated because of the data availability.
Randomization	All animals were maintained in the same environment and were randomly assigned to the experimental groups. For cell culture experiments, a single cell line was used and therefore randomization was not required. For human samples, randomization was not required since no experimental intervention was performed.
Blinding	For data automatically collected by instruments, such as flow-cytometry and immunoblotting, researchers were not blinded as observer bias is expected not to affect the results. For data manually collected by researchers, such as clinical score and bead number counting, researchers were blinded to group allocation during data collection.

Reporting for specific materials, systems and methods

We require information from authors about some types of materials, experimental systems and methods used in many studies. Here, indicate whether each material, system or method listed is relevant to your study. If you are not sure if a list item applies to your research, read the appropriate section before selecting a response.

Materials & experimental systems

n/a	Involved in the study
	[x] Antibodies
	[x] Eukaryotic cell lines
[x]	Palaeontology and archaeology
	[x] Animals and other organisms
[x]	Clinical data
[x]	Dual use research of concern

Methods

n/a	Involved in the study
[x]	ChIP-seq
	[x] Flow cytometry
[x]	MRI-based neuroimaging

Antibodies

Antibodies used

All antibodies used in this study are listed in the Supplementary Table 2.
anti-Sialyl-Tn ,clone MLS132, Source: FujiFilm Cat.# 010-25881
anti-cytokeratin7 , clone EPR17078 , Source: Abcam Cat.# ab181598
anti-mouse IgE, biotin , clone R35-118 , Source: BD Biosciences Cat.# 553419
anti-mouse IgG1, biotin , clone N/A (polyclonal) , Source: SouthernBiotech Cat.# 1070-08
anti-mouse IgG2a, biotin , clone N/A (polyclonal) , Source: SouthernBiotech Cat.# 1080-08
anti-mouse IgG, HRP , clone N/A (polyclonal) , Source: Cell Signaling Technology Cat.# 7076
anti-CD16/CD32 , clone 2.4G2 , Source: BD Biosciences Cat.# 553141
anti-CD11b, FITC , clone M1/70 , Source: BioLegend Cat.# 101206
anti-CCR3, PE , clone 83101 , Source: R&D SYSTEMS Cat.# FAB729P
anti-mouse I-A/I-E, PE , clone M5/114.15.2, Source: Biolegend Cat.# 107608
anti-CD45, PerCP/Cyanine5.5 , clone 30-F11 , Source: BioLegend Cat.# 103132
anti-CD45, biotin , clone 30-F11 , Source: BioLegend Cat.# 103104
anti-CD11c, PE/Cyanine7 , clone N418 , Source: BioLegend Cat.# 117318
anti-Siglec-F, Alexa Fluor647 , clone E50-2440 , Source: BD Biosciences Cat.# 562680
anti-Muc5ac , clone 45M1 , Source: Abcam Cat.# ab3649
anti-mouse AlexaFluor 647 , clone N/A (polyclonal) , Source: ThermoFisher Scientific Cat.# A21236
anti-mouse AlexaFluor 568 , clone N/A (polyclonal) , Source: ThermoFisher Scientific Cat.# A11031
anti-rabbit AlexaFluor 488 , clone N/A (polyclonal) , Source: ThermoFisher Scientific Cat.# A11034
anti-rabbit AlexaFluor 488 Superclonal, clone N/A (not disclosed), Source: ThermoFisher Scientific Cat.#A27034

Validation

All antibodies are commercially available and those specificities were tested by manufactures. All vendors and catalog numbers of antibodies are listed above and detailed information is available on the websites. For flow-cytometry analyses, specificity was evaluated using the proper control including isotype control antibody. For WB experiments, specificity was validated using desialylation for sialyl-Tn antibody.
Data sheets are available from the the web links as described below.
anti-Sialyl-Tn https://labchem-wako.fujifilm.com/jp/product/detail/W01W0101-2588.html
anti-cytokeratin7 https://www.abcam.com/cytokeratin-7-antibody-epr17078-cytoskeleton-marker-ab181598.html
anti-mouse IgE, biotin https://www.bdbiosciences.com/en-eu/products/reagents/immunoassay-reagents/elisa/biotin-rat-anti-mouse-ige.553419
anti-mouse IgG1, biotin https://www.southernbiotech.com/goat-anti-mouse-igg1-human-ads-biot-1070-08
anti-mouse IgG2a, biotin https://www.southernbiotech.com/goat-anti-mouse-igg2a-human-ads-biot-1080-08
anti-mouse IgG, HRP , https://en.cellsignal.jp/products/secondary-antibodies/anti-mouse-igg-hrp-linked-antibody/7076
anti-CD16/CD32 https://www.bdbiosciences.com/ja-jp/products/reagents/flow-cytometry-reagents/research-reagents/single-color-antibodies-ruo/purified-rat-anti-mouse-cd16-cd32-mouse-bd-fc-block.553141
anti-CD11b, FITC https://www.biolegend.com/ja-jp/lyophilized-control-cells/fitc-anti-mouse-human-cd11b-antibody-347
anti-CCR3, PE https://www.rndsystems.com/products/mouse-ccr3-pe-conjugated-antibody-83101_fab729p
anti-mouse I-A/I-E, PE, https://www.biolegend.com/ja-jp/products/pe-anti-mouse-i-a-i-e-antibody-367
anti-CD45, PerCP/Cyanine5.5 https://www.biolegend.com/ja-jp/productstab/percp-cyanine5-5-anti-mouse-cd45-antibody-4264?GroupID=BLG6829
anti-CD45, biotin https://www.biolegend.com/ja-jp/products/biotin-anti-mouse-cd45-antibody-98?GroupID=BLG1932
anti-CD11c, PE/Cyanine7 https://www.biolegend.com/ja-jp/products/pe-cyanine7-anti-mouse-cd11c-antibody-3086
anti-Siglec-F, Alexa Fluor647 https://www.bdbiosciences.com/en-eu/products/reagents/flow-cytometry-reagents/research-reagents/single-color-antibodies-ruo/alexa-fluor-647-rat-anti-mouse-siglec-f.562680
anti-Muc5ac https://www.abcam.com/mucin-5ac-antibody-45m1-ab3649.html
anti-mouse AlexaFluor 647 https://www.thermofisher.com/antibody/product/Goat-anti-Mouse-IgG-H-L-Highly-Cross-Adsorbed-Secondary-Antibody-Polyclonal/A-21236
anti-mouse AlexaFluor 568 https://www.thermofisher.com/antibody/product/Goat-anti-Mouse-IgG-H-L-Highly-Cross-Adsorbed-Secondary-Antibody-Polyclonal/A-11031
anti-rabbit AlexaFluor 488 https://www.thermofisher.com/antibody/product/Goat-anti-Rabbit-IgG-H-L-Highly-Cross-Adsorbed-Secondary-Antibody-Polyclonal/A-11034
anti-rabbit AlexaFluor 488 Superclonal https://www.thermofisher.com/antibody/product/Goat-anti-Rabbit-IgG-Heavy-chain-Secondary-Antibody-Recombinant-Polyclonal/A27034

Eukaryotic cell lines

Policy information about cell lines and Sex and Gender in Research

Cell line source(s)	HT29-MTX-E12 cell line was obtained from European Collection of Authenticated Cell Cultures (ECACC)
Authentication	None of the cell lines used were authenticated; the cells were directly obtained from ECACC.
Mycoplasma contamination	Cell cultures were free of Mycoplasma.
Commonly misidentified lines (See ICLAC register)	No commonly misidentified lines were used in this study.

Animals and other research organisms

Policy information about studies involving animals; ARRIVE guidelines recommended for reporting animal research, and Sex and Gender in Research

Laboratory animals	Wild-type C57BL/6J (B6J) mice and Balb/c mice were bred in-house or purchased from Sankyo Labo Service Corporation (Tokyo, Japan). Ao mice were generated by backcrossing the B6J mice to Balb/c mice. St6galnac1 knockout mice on the B6J background have been generated in the animal facility of Juntendo University. All mice used in this study were 5-9 weeks old.
Wild animals	No wild animals were used in this study.
Reporting on sex	Male and female mice were equally used for all experiments.
Field-collected samples	No field-collected samples were used in this study.
Ethics oversight	All animal experiments were approved by the Institutional Animal Care and Use Committee at Juntendo University (Approval numbers: 2022107 and 2020136) and performed in accordance with the Association for Research in Vision and Ophthalmology (ARVO) statement for the animal use in Ophthalmic and Vision Research.

Note that full information on the approval of the study protocol must also be provided in the manuscript.

Flow Cytometry

Plots

Confirm that:

- [x] The axis labels state the marker and fluorochrome used (e.g. CD4-FITC).
- [x] The axis scales are clearly visible. Include numbers along axes only for bottom left plot of group (a 'group' is an analysis of identical markers).
- [x] All plots are contour plots with outliers or pseudocolor plots.
- [x] A numerical value for number of cells or percentage (with statistics) is provided.

Methodology

Sample preparation	Conjunctival tissue was dissected, minced, and digested in RPMI1640 supplemented with 10% FCS, 1 mg/mL collagenase (FujiFilm, Japan) and 0.5 mg/mL DNase I with continuous stirring for 1 h at 37°C. The dispersed cell suspension was filtered through a nylon mesh. The lymph node cells were dispersed directly through a nylon mesh. The washed cells were blocked with anti-CD16/CD32 antibody (clone 2.4G2) for 10 min at 4°C. The cells were further incubated with surface-staining antibodies at 4°C for 20 min. Washed cells were resuspended in PBS containing 2% FCS and 1 µg/mL DAPI.
Instrument	BD FACSVerse flow cytometer
Software	Acquisition: BD FACSuite (1.0.6)<br>Analysis: Flowjo (10.8.1)
Cell population abundance	Sorting experiment was not performed on primary cells.
Gating strategy	Gating strategy is shown in Supplementary Fig. 8.

- [x] Tick this box to confirm that a figure exemplifying the gating strategy is provided in the Supplementary Information.
